# Acknowledgment to the Reviewers of *Foods* in 2022

**DOI:** 10.3390/foods12030445

**Published:** 2023-01-18

**Authors:** Foods Editorial Office

**Affiliations:** MDPI AG, St. Alban-Anlage 66, 4052 Basel, Switzerland

High-quality academic publishing is built on rigorous peer review. *Foods* was able to uphold its high standards for published papers due to the outstanding efforts of our reviewers. Thanks to the efforts of our reviewers in 2022, the median time to first decision was 16 days and the median time to publication was 37 days. Regardless of whether the articles they examined were ultimately published, the editors would like to express their appreciation and thank the following reviewers for the time and dedication that they have shown *Foods*:
Aamir IqbalKonstadinos MattasAamir ShehzadKonstantin V. MoiseenkoAaron K. HoshideKonstantin VolchoAarti BainsKonstantina TsikrikaAbbas RohaniKonstantinos ArsenopoulosAbdallah Fikry A. MahmoudKonstantinos ArvanitisAbdallah Tageldein MansourKonstantinos PapadimitriouAbdelfattah EL MoussaouiKorawan SringarmAbdel-Moneim EidKosuke MotokiAbdelnaser A. ElzaawelyKosuke NakamuraAbdelrahim HassanKoushik AdhikariAbdelraouf. M. AliKoutaro HasegawaAbdelsamed I. ElshamyKovačević Danijela BursaćAbdelsattar AbdelkhalikKresimir MastanjevicAbderrahman LamaouiKrishnamoorthy HegdeAbderrazek MaaroufiKrishnamoorthy RambabuAbdeslam Hassen MeniaiKriskamol Na JomAbdo HassounKristen E. GibsonAbdollah GhasemianKristen MatakAbdorreza Mohammadi NafchiKristian PastorAbdul BasitKristina KukurovaAbdul Hadi NabilahKristina MastanjevićAbdul QayyumKristina MenaAbdul RahamanKriton GrigorakisAbdul RohmanKrystyna RejmanAbdullah Al MamunKrystyna Szymandera-BuszkaAbdullah Antar SaberKrzystof LechAbdulrazaq YahayaKrzysztof B. BecAbdulwahed M. AboukarimaKrzysztof CiecielągAbdur RahmanKrzysztof DasiewiczAbha JainKrzysztof DziedzicAbhay K. PandeyKrzysztof PrzybyłAbhay TiwariaKrzysztof RutkowskiAbhinav DubeyKrzysztof SkowronAbhiram ArunkumarKsenija GasicAbiodun Olusola OmotayoKuan-Chen ChengAbiodun OmotayoKui ZhongAbolfazl AlirezaluKumarasamy MurukesanAbraham Méndez-AlboresKun YangAbraham Wall MedranoKunal PalAbu Khayer Md. Muktadirul Bari ChowdhuryKunal PratapAbul HossainKwang Won SeoAbulimiti YiliKwesi Saalia FiribuAchinta BordoloiKyoji MoritaAchmad SolikhinKyung Hwan JungAcosta Diego Fernando RoaKyung Taek RimAda Margarida Correia Nunes Da RochaL. M. MedinaAdam KlimowiczLacrimioara SenilaAdam KowalczykLadislav Eav KokoškaAdamantini KyriacouLaetitia ThéronAdela AbellánLai-Hoong ChengAdela Mena-MoralesLaila Muñoz-CastellanosAdele PapettiLaima ČesonienėAderval S. LunaLaís Mariano ZaninAdetiya RachmanLajnaf RouaAdil GaniLamilla ClaudioAdina FrumLan-Szu ChouAdina-Elena SegneanuLara CostantiniAdis PuškaLars WikingAditi PrabhakarLászló ÓzsváriAdnan AynaLászló SiposAdnan IsmaielLászló SzalayAdrian GrozeaLato PezoAdrian Krzysztof AntosikLaura BarpAdrián MatencioLaura CampanoneAdrián RabadánLaura Carmen ApostolAdrian RivisLaura CelanoAdrian ȘpacLaura Domínguez DíazAdriana ArigòLaura Dorina DinuAdriana Burlea-SchiopoiuLaura DugoAdriana Cunha NevesLaura EscriváAdriana DabijaLaura FariñaAdriana FarahLaura GazzaAdriana FilipLaura Martínez-CarrascoAdriana GrigorașLaura MitreaAdriana HernándezLaura RighettiAdriana MorarLaura SbarceaAdriana S. FrancaLaura Settier-RamírezAdriano Costa De CamargoLaura StanAdriano Gomes Da CruzLaure PoquetAdriene Ribeiro LimaLaurent BazinetAdy GiordanoLaurent DufosséAekkhaluck IntharuksaLaurianne ParavisiniAera JangLavinia BuruleanuAfaf Kamal El-AnsaryLeandro CardosoÁfrica SanchizLeanne YoungAfroditi ChatzifragkouLech AdamczakAgata BiadałaLecturer Carmen Rodica PopAgata Białecka-DębekLee-Ann JaykusAgata Carmela NicolosiLei QinAgata FabiszewskaLei RaoAgata GórskaLei WangAgata Malak-RawlikowskaLeif- Alexander GarbeAgata MarzecLeigh FrancisAgata Olszewska-WiddratLeilane Rocha Barros DouradoAgata PanethLeilson BezerraAgata SommerLeilson RibeiroAgata SwieciloLeiqing PanAgata Wojciechowicz-BudziszLélia ChambelAgata ZnamirowskaLenka CeslovaAggrey Pemba GamaLenka KouřimskáÁgnes AlbertiLenka VorlováAgnes Dienes-NagyLenuta-Maria SutaAgnes MwangwelaLeonard KoolmanAgnieszka Dobrzyńska-IngerLeonardo CasiniAgnieszka Ewa StępieńLeonardo OrnellaAgnieszka Ewa Wia̧cekLeonardo SepúlvedaAgnieszka FogelLeonardo TeixeiraAgnieszka HerosimczykLeontina C. GurguAgnieszka JasińskaLeontina LipanAgnieszka KicelLeshweni Jeremia ShaiAgnieszka KitaLesław JuszczakAgnieszka LatochLeszek KadzińskiAgnieszka LudwiczakLeszek SzablewskiAgnieszka NarwojszLeszek WanatAgnieszka NawrockaLeticia Diez-QuijadaAgnieszka OrkuszLeticia Xochitl Lopez-MartinezAgnieszka PiekaraLevent AydemirAgnieszka Pluta-KubicaLi DapengAgnieszka RichertLi ZhangAgnieszka StawarskaLía Noemí GerschensonAgnieszka SzewczykLiang RongrongAgnieszka Szlagatys-SidorkiewiczLiang-Yu ChenAgnieszka Tajner-CzopekLibor CervenkaAgnieszka ViapianaLicia PantanoAgnieszka Wiszniewska-ŁaszczychLidia MuscarielloÁgoston TemesiLídia PinheiroAgung SugiriLidia Stasiak-RóżańskaAgung WahyonoLidija JakobekAgustín AriñoLidija KozacinskiAhm Khurshid AlamLidija KozačinskiAhmad Adlie ShamsuriLidija SvečnjakAhmad Fairuz OmarLifang FengAhmad G. HegaziLígia Isoni AuadAhmad J. ObaidullahLih-Shiuh LaiAhmad JahanbakhshiLiisa LähteenmäkiAhmad Khusairi AzemiLili HeAhmad NawazLili JiaAhmad ZareiLili MatsAhmed A. Abd El-MaksodLiliana CepoiAhmed A. SalehLiliana G. FidalgoAhmed A. ZakiLiliana TudoreanuAhmed A. ZakyLiliana Vargas-MurgaAhmed AbdeenLiliya FedulovaAhmed AliLiliya S. LogoydaAhmed BadrLiming WuAhmed El GhorabLin SongAhmed GabrLina CossignaniAhmed HajibLina RaudonėAhmed M. Abd-ElGawadLina ŠernaitėAhmed MedianiLinda BesterAhmed NafisLinda LorenzAhmed R. A. HammamLinda MonaciAhmed R. SofyLindsay BrownAhmed RadyLindsey SchierAhmed SalehLinnea BärebringAhmed ShehabLinus Mabughi NyiwulAhmed TahaLipan LeontinaAhmed ZayedLiqiang ZouAhmet AydinLisa DeanAhmet Emre YaprakLisa GorskiAida Mihaela VasileLisa MarinelliAida Moreira da SilvaLisa Oehrl DeanAida TurriniLisa PilkingtonAidilla MubarakLisa TuppoAijun HuLisa-Maria CallAilton Cesar LemesLisbeth MehliAilyn FadriquelaLise KorstenAimei ZhouLisete PaivaAimin JiangLitao YangAinara LópezLiu FeiAinaz KhodanazaryLiudmyla L. TitovaAirong ZhangLivia PatrascuAivars BerzinsLixing WangAjaikumar KunnumakkaraLiya LiuAjjampura C. VinayakaLiyuwork Mitiku DanaAkath SinghLjilja TorovićAkhilesh Kumar VermaLjubica DokićAkiko MizokamiLjubodrag VujisićAkira WanikawaLoh Teng-Hern TanAkmal NazirLoleny TavaresAlaa A. GaafarLolita TomsoneAlaa El-Din A. BekhitLong ChenAlaa Jabbar Abd Al-ManhelLong ShengAlaa KamelLong WuAlain D'AstousLongtao ZhangAlam Md BadrulLongyan ZhaoAlam ZebLoredana DumitrascuAlbert E. ParkerLoredana MarinielloAlbert KoulmanLorena ButinarAlbert Linton CharlesLorena MartínezAlbert T. PoortingaLorenzo GuzzettiAlberto A. Escobar-PuentesLorenzo MorelliAlberto Baños ArjonaLorenzo PalombiAlberto BarbiroliLorenzo StrattaAlberto GallegosLoreta BasinskieneAlberto Horcada IbáñezLoris PintoAlberto TondaLouis Kuoping ChaoAlberto VergaraLouis Wy LiuAlberto VitaliLouise JankAlcina MoraisLouise ManningAldo LaganàLoulouda BosneaAldo TavaLouwrens C. HoffmanAlejandra Acevedo FaniLuan Luong ChuAlejandra Garcia-AlonsoLuana Beatriz Dos Santos NascimientoAlejandra Rodriguez-HaralambidesĽubica RumanovskáAlejandra UrtubiaLubomir LapcikAlejandro A. TapiaLuboš SmutkaAlejandro Miguel Figueroa-LópezLuc NegroniAlejandro Pérez LariosLuc SaulnierAlejandro PlascenciaLuca AltamoreAlejandro Plascencia PlascenciaLuca Domenico D'AndreaAlejandro SanzLuca FasolatoAlejandro Téllez-JuradoLuca FioraniAleksandar DjordjevicLuca FontanesiAleksandar PetrovićLuca IseppiAleksandar Z. FištešLuca LaghiAleksandar Ž. KostićLuca LanotteAleksandr KazachenkoLuca RosciniAleksandra A. JovanovićLuca SettanniAleksandra C. ArsićLuca ValentiniAleksandra Duda-ChodakLucas Louzada PereiraAleksandra GavaricLucia CavigliAleksandra KocotLucia De LucaAleksandra KołotaLucia Maria Carareto AlvesAleksandra KowalskaLucia MorroneAleksandra KristoLucia ParafatiAleksandra Maria StaszakLucía Seguí GilAleksandra MišanLucia SepeAleksandra NesicLucía SoliñoAleksandra Nikolić-KokićLucia VanniniAleksandra PawlaczykLucian CopoloviciAleksandra SzydlowskaLuciana De Siqueira OliveiraAleksandra Tepić HoreckiLuciana Di GiacintoAleksandra TorbicaLuciana MandrioliAleksandra WilczyńskaLuciana Ruschel Dos SantosAleksandra ZambrowiczLuciana SolerAleksei KaledaLuciana TorquatiAlemayehu (Alex) AmbawLuciano PinottiAlena SalákováLucie DrábováAlena StuparLucio CappelliAles RajchlLucio CapursoAleš TomčalaLucyna DymińskaAlessandra BaldiLudmila KaramonovaAlessandra BiancolilloLudmila RudiAlessandra BordoniLuigi BottaAlessandra Del CaroLuigi CastaldoAlessandra FratianniLuigi Di BitontoAlessandra JagerLuigi MenghiniAlessandra PalladiniLuigino BarisanAlessandra RivaLuís AbrunhosaAlessandro BonadonnaLuis Alfonso Trujillo-CayadoAlessandro Di CerboLuis Angel Medina JuarezAlessandro GrinzatoLuis Arturo Bello-PérezAlessandro RobertielloLuís C. N. Da SilvaAlessandro SeguinoLuis Daniel Goyzueta MamaniAlessandro ZappiLuis F. BarbisanAlessia CarianiLuis F. Luna ReyesAlessia FazioLuis Federico CasassaAlessia LevanteLuís Filipe-RibeiroAlessia TropeaLuis G. Sequeda-CastañedaAlessio AllegraLuis Guillermo Gonzalez OlivaresAlessio CappelliLuis Henrique Souza GuimarãesAlessio MassironiLuís Kluwe De AguiarAlex López-CórdobaLuis López-GiraldoAlex MartynenkoLuis M. Carrillo-LópezAlex OkaruLuis Miguel EsparzaAlexander A. KirillovLuis MojicaAlexander FjældstadLuis NogueraAlexander G. HaslbergerLuis Noguera ArtiagaAlexander GovarisLuís PatarataAlexander K. GoroncyLuís PintoAlexander KasumyanLuis Puente-DiazAlexander O. ChizhovLuis RodrigoAlexander SidorenkoLuísa C. RodriguesAlexander ToetLuisa PilanAlexandr KazachenkoLuisella VerottaAlexandra BarboutiLuis-Felipe GutiérrezAlexandra JitǎreanuLuiz A. ColnagoAlexandra LianouLuiz Carlos Correa FilhoAlexandre LamasLukas ParkerAlexandru RusuŁukasz BobakAlexandru Vasile RusuŁukasz DąbrowskiAlexandru VasincuŁukasz K. KaczyńskiAlexandru VlaicuŁukasz ŁopusiewiczAlexey OrlovŁukasz MigdałAlexey ShevelevŁukasz SeczykAlexios A. AlexopoulosŁukasz SzeleszczukAlexsandr ProsekovŁukasz TomczykAlfonso M. VidalŁukasz WajdaAlfredo Ayala-AponteLuminita CrisanAlfredo J. IbáñezLuna Maslov BandićAlfredo MontañoLunjie HuangAlfredo TeixeiraLu-Sheng HsiehAlfredo Vázquez-OvandoLutfiye Yilmaz-ErsanAli AberoumandLuz H. Villalobos-DelgadoAli AkbarLuz Indira Sotelo-DíazAli BoolaniLuz María Paucar-MenachoAli BougatefLyda G. GarciaAli CharkheshtLydiane NabecAli DadM. A. Del NobileAli HeshmatiM. Belal HossainAli ImranM. Carmen SeijoAli JafarpourM. Elvira López-CaballeroAli KhanjariM. Eugenia TornadijoAli MoayediM. G. SemenovaAli SaeedM. JameelAli Sedaghat DoostM. Leonor NunesAli UbeyitogullariM. Margarida Baleiras-CoutoAlice MarciniakM. R. MohammadabadiAlice VilelaM. R. MozafariAlicia Gil-RamírezM. T. MasturaAlicia Grajales-LagunesMabrouk ElsabaghAlicja AurigaMacello MasciniAlicja PonderMaciej GuzikAlida SorrentinoMaciej JarzebskiAlifdalino SulaimanMaciej KaczmarskiAlina CuletuMaciej NastajAlina MihailovaMaciej OziembłowskiAlina Violeta UrsuMaciej TankiewiczAline Camarão Telles BiasotoMaciej WłodarczykAline Pereira De OliveiraMack ShelleyAline T. TociMadalena Vieira-PintoAliona Ghendov-MosanuMadalina SandulescuAlireza SadeghiMaddalena ParafatiAlireza SanaeifarMadhu KamleAlireza SeidaviMaegala Nallapan Nallapan ManiyamAlirica SuarezMagdalena BartnikAlisa RudnitskayaMagdalena DadanAlison GrayMagdalena Franczyk-ŻarówAliyar FouladkhahMagdalena Frej-MądrzakAliyu MohammadMagdalena GantnerAljaz MedicMagdalena Jeszka-SkowronAllah DittaMagdalena KrauzeAlma D. Alarcon-RojoMagdalena KrystyjanAlma Delia Alarcon-RojoMagdalena LigorAlma R. Islas-RubioMagdalena MititeluAlmira RamanavicieneMagdalena MontowskaAlmudena Marrufo-CurtidoMagdalena PodbielskaAlmudena V. MerchanMagdalena Polak-ŚliwińskaAltynai SharipovaMagdalena ScheibeAlvaro BicudoMagdalena Śmiglak-KrajewskaAlyexandra ArienzoMagdalena SobocińskaAlyssa Hidalgo VidalMagdalena Wirkowska-WojdyłaAlžbeta HegedűsováMagdalena WójciakAmalia ConteMagdalena Wróbel-KwiatkowskaAmalia PiscopoMagdalena WronaAmalio Santacruz VarelaMagdalena ZalewskaAman DurejaMagdalena Zielińska-DawidziakAmanda GendersMaha HoteitAmanda J. DeeringMahadi Hasan MasudAmanda KinchlaMaheen GullAmanda SiegelMahendran RadhakrishnanAmanda StewartMahipal Singh KesawatAmanda V. SardeliMahmoud AbughoushAmani ChroudaMahmoud AlagawanyAmani TaamalliMahmoud Amouzadeh TabriziAmanullah KhanMahmoud AzzamAmbrogina AlbergamoMahmoud BendaryAmeer Fawad ZahoorMahmoud El-MaghrabeyAmeer KhusroMahmoud F. SeleimanAmel TaibiMahmoud GhasemnezhadAmelia DelgadoMahmoud MadkourAmérica Chávez-MartinezMahmoud Mostafa AzzamAmin Mousavi KhaneghahMahnaz EstekiAmin N. OlaimatMahsa TabariAmina RhouatiMaik A. JochmannAmir Larki HarcheganiMaimunah Mohd AliAmit JaiswalMaja BasicAmjad HameedMaja BenkovićAmmar Al-FargaMaja BulatovicAmmar AltemimiMaja Jazvinšćak JembrekAmmar BaderMaja KaramanAmna SaharMaja Krstić RistivojevićAmr AminMaja LeitgebAmr FaroukMaksim RebezovAna A. Vilas-BoasMalco Cruz‐RomeroAna AlimpicMalcolm RileyAna AriasMałgorzata BiałekAna BarrosMałgorzata Brzezińska-RodakAna Belén Díaz SánchezMałgorzata CzerwonkaAna Belén RoperoMałgorzata DżuganAna C. GonçalvesMałgorzata Ewa DrywieńAna CastanhoMałgorzata GniewoszAna Clara AprotosoaieMałgorzata GrabarczykAna Cristina Agulheiro SantosMalgorzata GrembeckaAna Cristina Sanchez-GimenoMałgorzata Kalemba-DrożdżAna Elisa RatoMalgorzata KorzeniowskaAna Estela LedesmaMałgorzata Kosicka-GębskaAna FariaMalgorzata KosteckaAna Filipa PiresMałgorzata KozyraAna GavrilovićMałgorzata KrzywonosAna Gomes-BispoMalgorzata KucinskaAna HranilovicMałgorzata Lasik-KurdyśAna I. CarrapisoMałgorzata MoczkowskaAna Isabel CostaMałgorzata Moczkowska-WyrwiszAna JeromelMalgorzata NowackaAna Jiménez CantizanoMałgorzata PawlosAna Jurinjak TusekMałgorzata PiecykAna KaicMałgorzata RobakAna Karine F. De CarvalhoMałgorzata RutkowskaAna L. Rentería-MonterrubioMałgorzata Schlegel-ZawadzkaAna Laura De La GarzaMałgorzata SierockaAna LeahuMałgorzata SobczakAna Lúcia FerreiraMałgorzata StarowiczAna Lucía Morocho-JácomeMałgorzata Szultka-MłyńskaAna M. FerreiraMałgorzata TabaszewskaAna Margarida SilvaMałgorzata WroniakAna María BotanaMalgorzata Zakłos-SzydaAna María Calderón De La BarcaMałgorzata ZiarnoAna María Jiménez CarveloMalinee SriariyanunAna María RojasMallikharjuna KoriviAna Marija Jagatić KorenikaMalshani L. PathirathnaAna MornarMamoru IsemuraAna MucaloManat ChaijanAna Paula DelamareMandana BimakrAna Paula RebellatoMandana PahlavaniAna Pinto De MouraManeka MalalgodaAna Rodríguez Bernaldo De QuirósManel IssaouiAna S. LuisMan-Gi ChoAna Sanches SilvaManikandan MuthuAna Silvia PrataManikantan PappuswamyAna TomićManish KumarAna VinhaManju BalaAnabel López OrtizManohar Prasad BhandariAnabela RaymundoManoj GhasteAnamaria PopManoj KumarAnamaria SdrobisManoj Kumar TripathiAna-Maria TănaseManoj MahawarAnand Babu PerumalManol OgnyanovAnargyros MoulasManolis LadoukakisAnastasia D. PournaraMansoore ShamiliAnastasia KyriakoudiMansureh GhavamAnastasia SemenovaManuel SánchezAnastasia VenierakiManuel ValeroAnastasios NikolaouManuel VenturiAnatolii KucherManuel Viuda-MartosAnatoly P. SobolevManuela GiordanoAnatoly V. ZherdevManuela GrimaldiAnayansi Escalante-AburtoManuela GuerraAnca Corina FarcasManuela PanićAnders H. KarlssonManuela Vaz-VelhoAnderson Ferreira Da CunhaManuela ZadravecAnderson Junger TeodoroMaqsood MalikAndi DirpanMarc A. BealAndras JungMarc MarescaAndré DelavaultMarc RegierAndré Fioravante GuerraMarcel KrzanAndré LipskiMarcela C. FerreiraAndre Ricardo AlcardeMarcela Lilian MartinezAndré Rolim BabyMarcella AvondoAndrea BellincontroMarcello BioccaAndrea BragaglioMarcello StancoAndrea BrandoliniMarcello TagliaviaAndrea BuneaMarcelo FranchinAndrea DidierMarcelo Paes BarrosAndrea GarmynMarcelo RosminiAndrea Herrero-CerveraMarcelo Valle GarciaAndrea HoehnelMarcia NitschkeAndrea MachyňákováMarcia Regina AssalinAndrea ManciniMarcin Gra̧ZAndrea Maria GuarinoMarcin KadejAndrea PautzMarcin KidońAndrea RagusaMarcin LukasiewiczAndrea VersariMarcin SchmidtAndrea WangorschMarcio SchmieleAndrea Y. Guadarrama-LezamaMarco A. MurgiaAndreas EisenreichMarco AcciaroAndreas HåkanssonMarco Antonio TrindadeAndreas KoschellaMarco ConsumiAndreea RatiuMarco CullereAndreea-iren SerbanMarco Dalla RosaAndrei BorșaMarco FaietaAndrei HonciucMarco FornasierAndrei SteindorffMarco IammarinoAndreia FreitasMarco PoianaAndreia RibeiroMarco RinaudoAndreia RodriguesMarcos David FerreiraAndrej KirbišMarcos FloresAndreja GoršekMarcos NanniAndreja Leboš PavuncMarcus DymondAndres Barajas-SolanoMarcus SchmidtAndrés J. RascónMarcus ScottiAndresa GomesMarek AljewiczAndrew Bamidele FalowoMarek BobkoAndrew HungMarek CebratAndrew RosenthalMarek GancarzAndrew SpringMarek KulbackiAndrew SzilagyiMarek MarkowskiAndrey A. ShelenkovMarek ProchazkaAndrey A. TyuftinMarek SikorskyAndrey MarchevMarek ŠnircAndrey NagdalianMarek TobiszewskiAndriati NingrumMargaret WeinrothAndrii MaryninMargarita GarcíaAndrija CiricMargherita CaccamoAndrius GarbarasMargherita GavagninAndriy SynytsyaMari H. OgiharaAndriy VoronovMaría A. F. FerrusAndruta MuresanMaria A. HorstAndrzej AndersMaria A. LongoAndrzej BryśMaria Aderuza HorstAndrzej CieslikMaria Alice Zarur CoelhoAndrzej OkruszekMaría Ángeles SantosAndrzej PółtorakMaria Anna MaggiAneta BrodziakMaría Antonia Alvarez FernandezAneta CegiełkaMaria Antonietta CiardielloAneta PaterMaria AponteAneta PopovaMaria AspriAneta SławińskaMaria AtanassovaAngel CobosMaria Barbara RóżańskaAngel Josabad Alonso-CastroMaria Belén LinaresAngela FabianoMaria Belen VignolaAngela FaddaMaria BrassescoÁngela G. SolaesaMaria C. GiannakourouAngela Jedlovszky-HajduMaria CalassoAngela R. PiergiovanniMaria CamposAngela RacioppoMaria Carla CraveroAngela SantilioMaria Carolina OtáloraAngela SorboMaria CavalettoAngela SorrentinoMaria Cecilia PuppoAngela StortiniMaria CefolaAngela TarabellaMaria Ciudad-MuleroAngélica Villarruel-LópezMaria Concepcion MartinezAngélique Nicolas MessiMaría Concepción Parra-MeroñoAngelo CavallieriMaria Da Graça Costa MiguelÂngelo Luiz Fazani CavallieriMaria De La Luz ZambranoAnia CraveroMaría De Lourdes García MagañaAnicuta StoicaMaría Del Carmen PérezAnil K. VermaMaria Del Refugio Castañeda-ChavezAnil Kumar PatelMaria DermikiAnil Kumar SirohaMaria DigiacomoAnil VermaMaria DimopoulouAniruddha SarkerMaria Do Carmo PeluzioAnis Ben HsounaMaria Dolors GuàrdiaAnita MilićMaría Dolors Parés-OlivaAnita PichlerMaria Drussilla BuscemiAnita Silvana Ilak PeršurićMaria DzikućAnita SpychajMaria E. AmaralAnja JoachimMaría E. García-PastorAnja Steffen-HeinsMaria Eduarda Machado AraújoAnka Trajkovska PetkoskaMaria Eduarda PotesAnkit JhaMaría Elena González-FandosAnna ArnoldiMaria Elena Sosa MoralesAnna Berthold-PlutaMaria Eleni SakavitsiAnna CarboneMaría Emilia SantillánAnna Czajkowska-KośnikMaría Esther Ortega-CerrillaAnna DiowkszMaría Eugenia Jaramillo FloresAnna FlorowskaMaria Federica TrombettaAnna GrygierMaria Fekete FarkasAnna Irene De LucaMaría Fernández-LobatoAnna JakubczykMaria Filomena BarreiroAnna Jarosz-WilkołazkaMaria Francesca IuliettoAnna Kamińska-DwórznickaMaria FranciosiniAnna KasprzykMaria Fredriksson-AhomaaAnna Kiełtyka-DadasiewiczMaria FreitasAnna KołtonMaría GianelliAnna Kostecka-GugalaMaria Grazia MelilliAnna LanteMaria GrzegorzewskaAnna ŁepeckaMaría Isabel SaezAnna M. HawryłMaria J. AlcaldeAnna M. R. HayesMaría J. BenitoAnna MadejskaMaría Janeth Rodríguez-RoqueAnna Maria PappalardoMaría Jesús Serrano AndrésAnna MilczarekMaria João BarrocaAnna Muzykiewicz-SzymańskaMaria João CabritaAnna N. BerlinaMaria João RamalhosaAnna NowakMaria Jorge Geraldes CamposAnna OgrodowczykMaría José Aliaño GonzálezAnna OnopiukMaría José Beriáin ApesteguíaAnna Paula Azevedo De CarvalhoMaría José Muñoz-AlférezAnna Picinelli-LoboMaría José ValeraAnna Przybylska-BalcerekMaria KatsouliAnna PtaszekMaria LavillaAnna Rafaela Cavalcante BragaMaria Leonor FaleiroAnna RealeMaria Leonor NunesAnna Rita BiliaMaria LeporeAnna Rybarczyk-PlonskaMaria LopesAnna SadowskaMaria Lucia Valeria De ChiaraAnna SandionigiMaria Luisa AmodioAnna SkicMaría Lujan FerreiraAnna StójMaria Madalena RinaldiAnna Szczerba-TurekMaria MaistoAnna Wajs-BonikowskaMaria MartuscelliAnna Winiarska-MieczanMaria MastorakiAnna WirkijowskaMaría Mora GijónAnna ZamoraMaria NobileAnna ŻbikowskaMaria Olga VarráAnna Zielińska-ChmielewskaMaria PaciulliAnna Zimoch-KorzyckaMaría Perez-JiménezAnna ŻołnierczykMaría Pilar García MonteroAnnalisa ChiavaroliMaria RâpăAnnalisa De BoniMaria Raquel Ventura LucasAnnalisa RicciMaría RivasAnnamaria GerardinoMaria RomoAnnarita PanighelMaría SerranoAnne TeterMaría Shantal Rodríguez-FloresAnne-Marie HermanssonMaria Sonia AlmeriaAnne‐Marie ReißnerMaria Stella CosioAnnette BrandtMaria Teresa FrangipaneAnnette KuehnMaría Teresa Murillo-ArbizuAns EilanderMaria TurtoiAnssi ÖörniMaria Valderez Ponte RochaAnte GalicMaría Verónica BaroniAnte LončarićMaria Victoria AlvarezAnte PrkićMaría Victoria SarriésAnthia MatsakidouMariabeatriz GlóriaAnthimia BatrinouMariadoss Arokia Vijaya AnandAnthony HutinMariaelena Di BiaseAnthony QuéroMarian ButuAnthony SalibaMarian RizovAntía Lestido-CardamaMariana A. FernándezAntoanela PatrasMariana Atena PoianaAnton FicaiMariana Buranelo EgeaAnton GáplovskýMariana María HuertaAntonela Ninčević GrassinoMariana S. CastroAntonella CostaMariana S. LinguaAntonella CostantiniMarianna GalloAntonella GiarraMarianna KocsisAntonella PasqualoneMarianna MartinelloAntonello SantiniMarianna OteriAntoni PlutaMarianna RaczykAntoni SzumnyMarianne MolinAntonia Dolores Asencio MartinezMariano Martínez VázquezAntonietta BaianoMariano StornaiuoloAntonín PřidalMariany Costa DepráAntonina ShumakovaMariarosaria SimeoneAntonio Bernabé-AntonioMaria-Theresia GekenidisAntonio BevilacquaMaribel Ovando MartínezAntonio CamaraMaricarmen Iñiguez-MorenoAntónio CardosoMaricica StoicaAntonio CillaMarie AlmingerAntonio ColettaMarie WalshAntonio De Jesús Cenobio-GalindoMarie-Alice FraitureAntonio Di MartinoMarie-Françoise SamsonAntonio Diogo Silva VieiraMarieke E. BruinsAntonio G. OliveiraMariela PatrignaniAntonio Garrido-FernándezMarie-Louise ScippoAntonio Gonzalez-CasadoMarieta L. C. PassosAntonio Lozano-LeónMarija Badanjak SabolovićAntónio M. PeresMarija BanožićAntonio MincioneMarija BoskovicAntonio MolinoMarija IvanovAntónio P. L. MartinsMarija KovačAntonio PanebiancoMarijana AčanskiAntonio PigaMarijana DjordjevićAntonio R. JimenezMarijana Marek SarićAntonio SecciaMarijana Zovko KončićAntonio SpanòMarilena AntunesAntonio StasiMarilena MuragliaAntonio TirelliMarilú A. Silva-EspinozaAntonio VioliMarina CerusoAntonios E. KoutelidakisMarina DaltoéAntonius IndartoMarina De Nadai Bonin GomesAntony C. CalokerinosMarina FidelisAnu HopiaMarina HolyavkaAnubhav Pratap SinghMarina JovanovićAnuj KumarMarina KostićAnusha PenumartiMarina MeflehAnwar UsmanMarina PapadeliAoki NoriakiMarina SokovicApinun KanpiengjaiMarina Svetec MiklenićApolonia SieprawskaMarina TomaševićApolonio Vargas-TorresMarina TomićArafat Abdel Hamed Abdel LatefMarina VillalonArantzazu Valdés GarcíaMarina VukojeAreej MerhiMarinella De LeoArfat AnisMario AmatoAriadnna Cruz-CórdovaMario D’AmicoArianna DickMario E. Rodríguez GarcíaAriel Colaço De OliveiraMario H. BarrosArifin Budiman NugrahaMário José Baptista FrancoArijit NathMario MikulaAris GiannakasMario ŠčetarArisekar UlaganathanMario SprovieriAristeidis TsagkarisMariola Koszytkowska-StawińskaArkadiusz ArtyszakMariola KozłowskaArkadiusz MatwijczukMariola M. BłaszczykArkadiusz SzpicerMarion Zammit MangionArlei ColdebellaMarios G. KostakisArlete Mendes FaiaMarios MataragasArmando A. Rayas-AmorMarisa PalazzoArmando CaseiroMaristella GussoniArmando J. D. SilvestreMarius Emil RusuArmando VenancioMariusz KucharskiArmando ZarrelliMariusz MojzychArmandodoriano BiancoMariusz RudyArmida Sánchez-EscalanteMariusz ŚlachcińskiArmin Mirzapour KouhdashtMariusz TichoniukArnoldo LopezMariusz WitczakArockiasamy Arun MiltonMariza LandgrafÁron TörökMark A. SmithÁrpád AmbrusMark SolomonArpita RoyMark W. SumarahArrigo CiceroMarko B. RadovićArta KronbergaMarko JukićArthitaya Kawee-aiMarko KaroglanArthur Bernardes Cecílio-FilhoMarlon DalmoroArtur GłuchowskiMarlous FockerArtur MartinsMarquez-Rocha Facundo-JoaquinArtur Roig-SaguésMarta ArczewskaArtur SzwengielMarta BertolinoArtur WiktorMarta ChmielArturo Gerardo Valdivia-FloresMarta CiecierskaArturo Hardisson De La TorreMarta CitelliArulkumar NagappanMarta Ferreiro-GonzálezArun GokulMarta Filipa SimõesArun K. SinghMarta Jeruszka-BielakAruna DhathathreyanMarta KadłubekArunaksharan NarayanankuttyMarta KopańskaArunas StirkeMarta OleszekArunkumar KarunanidhiMarta PasławskaArunkumar RanganathanMarta PlichtaArup DasMarta SajdakowskaArwa MustafaMarta Tomczyńska-MlekoArzu Kavaz YükselMartha Graciela Ruiz-GutiérrezArzu UçarMartin A. MasuelliAsaad Rehman Saeed Al-HilphyMartín AlujaAsabuwa Ngwabebhoh FahanwiMartin CaraherAsad NawazMartin Esqueda-ValleAsad UllahMartin GandAsada LeelahavanichkulMartin GottelandAshenafi F. BeyiMartin Krøyer RasmussenAshish M. MohiteMartin PecAshok Kumar MalikMartin RasporAshok Kumar TiwariMartin SenzAshot SargsyanMartín Valenzuela-MelendresAshutosh BahugunaMartina BednářováAsima ShahMartina CofeliceAsma AmlehMartina FoschiAsma AnsariMartina GrdišaAsma KhoobiMartina LudewigAsmaa Al-AsmarMartina PeršeAsmaa Galal-KhallafMartina PšenkováAsmaa KhafagaMartina PuccinelliAsta TamulevičienėMartina Srajer GajdosikAstrid BuicaMartina TorricelliAswin VerhoevenMartins SabovicsAswir Abd RashedMartis GeorgianaAtanas PavlovMartyna Lubinska-SzczygełAtef M. K. NassarMartyna MroczyńskaAthanasia GoulaMartyna WieczorekAthanasia O. MatemuMartyna Zagórska-DziokAthanasios K. LadavosMarwa A. A. FayedAthanasios KimbarisMarwa AliAthanassios AlexopoulosMarwa IssaAthula AttygalleMarwan E. MajzoubAtique-Ur RehmanMary Ellen CamireAtiyeh MahdaviMary GraceAtso RaasmajaMaryam Azizi LalabadiAttila GereMaryam RanjbarAttila JámborMaryam SalamiAttila OmbódiMaryke Labuschagne‪Attilio MateraMarysol AlvearAugustin C. MotMarzena Jeżewska-ZychowiczAunchalee AussanasuwannakulMarzena TomaszewskaAurelia Cristina NechiforMarzena Włodarczyk-StasiakAurora SilvaMarzena ZającAvik MukherjeeMarzia InnocentiAvtar SinghMarzia PezzolatoAwadhesh KumarMarzia VasarriAyaka KotemoriMarzieh HosseininezhadAyat F. HashimMasafumi NodaAyesha MurtazaMasahiko TsuchiyaAyesha SarkerMasahiro AkiyamaAyesha TahirMasanori AritaAyman MahmoudMasao YamasakiAyokanmi OreMasashi UemaAyoyinka O. OlojedeMasataka WakayamaAyse DundarMasayo KushiroAyse KoseMasayoshi MatsuiAyşegül ErdoğanMashiar RahmanAzadeh NilghazMasoumeh BejaeiAzalia Avila-NavaMassimiliano Matteo PellegriniAzat GabdulkhakovMassimo D’ArchivioAzeem IntisarMassimo LucariniAzilah AjitMassimo MozzonAzis SitanggangMassimo OnorAziz A. FallahMat Amin AmizaAzzurra StefanucciMatej PospiechB. WróblewskaMateus Henrique PetrarcaBabak PakbinMateusz JackowskiBabatunde OlawoyeMathew Hoani CummingBala Krishna PrabhalaMathieu SchwartzBalachandar VellingiriMatias SanchoBalamuralikrishnan BalasubramanianMatilda Steiner-AsieduBalarabe IsmailMatilde TuraBalasubramani Subramani ParanthamanMatina V. AngelopoulouBalasubramanian MalaikozhundanMatteo Della PortaBalenahalli RameshMatteo PeriniBalwinder SoochMatteo RamazzottiBambang KuswandiMatteo StoccheroBancha YingngamMatthaios P. KavvalakisBanu C. IulianaMatthew B. McsweeneyBanu KoçMatthew TaylorBanu Y. EkrenMatthias EderBaoye HeMatthias SipiczkiBárbara BiduskiMatthias UllrichBarbara BorczakMattia Di NunzioBarbara GiordaniMattia RapaBarbara GiussaniMattia SpanoBarbara Hawrylak-NowakMaud BonatoBarbara Krochmal MarczakMaud LangtonBarbara La GattaMaurice MonjereziBarbara ŁataMauricio Opazo-NavarreteBárbara Nieva-EchevarríaMauricio Redondo-SolanoBárbara Socas-RodríguezMaurizio MulasBarbara SokołowskaMauro MartínezBarbara SozańskaMauro RossiBarbara TeixeiraMauro Sergio Ferreira SantosBarbara TiozzoMaxim BurkinBarbaros ÖzerMaxim L. KuznetsovBarry LavineMaxwell Mewa-NgongangBarry ParsonsMaya M. ZaharievaBartłomiej GrygorcewiczMayra Aguilar-ZárateBartłomiej ZieniukMd Abdul WazedBartosz KruszewskiMd Nur HossainBartosz PiechowiczMd. Areeful HaqueBartosz SzulczyńskiMd. Meftaul IslamBasharat Nabi DarMecit Halil OztopBaskaran Stephen InbarajMeehee ChoBassem A. SabryMegan L. FalsettaBayu Adhi TamaMeghna GuptaBázár GyörgyMehdi Mogharabi-ManzariBeata BiernackaMehdi NikooBeata BilskaMehdi TrikiBeata DruzynskaMehmet OzcanBeata JanoszkaMehmet YilmazBeata KaletaMehri HadinezhadBeata KuczyńskaMeksianis NdiiBeata MikołajczakMelania GiammarcoBeata OlasMelinda FogarasiBeata WysokMelissa J. BentonBeatriz BrenerMelvin A. PascallBeatriz GullonMelvin R. Tapia-RodríguezBeatriz IsabelMemnune SengulBeatriz LimaMeng-I (Marie) KuoBeatriz QuiñonesMengmeng GuoBeatriz Sánchez-CalvoMengquan YangBeatriz Sevilla-MoránMenno Van Der VoortBegoña PaneaMerajuddin KhanBehnam Asgari LajayerMercedes Alfonso-PrietoBehzad KavianiMercedes G. LópezBeiwei ZhuMercedes Vázquez-EspinosaBekir TufanMerve KeskinBéla FiserMesfin TadesseBelem BarbosaMesut TaşkinBelinda López-GalánMeta MahendradattaBelur R. LokeshMeta SternišaBendegul OkumusMette Krogh LarsenBenedetta BottariMewa NgongangBeng Fye LauMian Abdur Rehman ArifBengi UsluMian Anjum MurtazaBengü ÖztürkMiaoyun LiBeniamino Cenci GogaMicaela ÁlvarezBeniamino T. Cenci-GogaMicha HoracekBenjamin M. BohrerMichael AppellBenjamin RocheMichael Brysch-HerzbergBenjamin ZeebMichael ChatzidimopoulosBenoît IgneMichael E. VaydaBerker NacakMichael G. KontominasBernadett LangóMichael G. WellerBernadette Emoke TelekyMichael GaenzleBernal-Mercado Ariadna ThalíaMichael HellwigBernard ChenMichael JahnckeBernard FayeMichael NetzelBernard MaringgalMichael PoteserBernardo RibeiroMichael TilleyBernd HitzmannMichael TunickBerta Baca-BocanegraMichael WeichhausBerta VázquezMichaela HavrlentováBettina SchwarzingerMichaela PatilaBeyza GultekinMichaela Schwaiger-HaberBeyza UlusoyMichail KmiecikBezon KumarMichail MichailidisBhagavathi SundaramMichailidis MichailBhagavathi Sundaram SivamaruthiMichał DrożdżekBhakti TannaMichał GazdeckiBianca CastiglioniMichał GóreckiBianca FoxMichał KrzyżaniakBianca R. AlbuquerqueMichał OczkowskiBibiana Juan GodoyMichalak-Tomczyk MagdalenaBijan AbadiMicheál O’MahonyBijl EtskeMichel Yves MistouBikramaditya GhoshMichela ContòBilige MengheMichela VerniBiljana LoncarMichele D'AmbrosioBiljana RabrenovićMichele FacciaBimal GhimireMichele PedrottiBimal Kumar GhimireMichelle ColgraveBinaya Bhusan NayakMichelle Ji Yeon YooBing-Lan LiuMiguel A. Aguilar-GonzálezBini WangMiguel A. AndrésBinod K. YadavMiguel A. PrietoBinosha FernandoMiguel Ángel González-CurbeloBiresaw Demelash AberaMiguel Ángel LópezBirsen YilmazMiguel Ángel Martínez‐TéllezBirthe P. Møller JespersenMiguel Ángel PrietoBlanca E. Morales-ContrerasMiguel Ángel Sogorb SánchezBlanca Hernández-LedesmaMiguel FariaBlanca Riquelme GallegoMiguel Portillo-EstradaBlanka ŠvecováMiguel PrietoBlaž StresMiguel Rebollo-HernanzBlaženka KosMiguel ViñasBo WangMihaela Aida VasileBo YuanMihaela Cristina BaicanBo ZhangMihaela SaracilaBogdan KontekMihaela SkrtBogdan SlencuMihaela TociuBogdan StępieńMihai TodicăBogumil BryckiMi-Jeong KimBohdan LuhovyyMi-Jung ChoiBojan MatkovskiMike PeckBojan ŽunarMike SissonsBojana BalančMikhail K. BeklemishevBojana DanilovicMikhail M. Vorob'EvBojana KokićMiklós MézesBojana ŠarićMiklós NagyBojana V. FilipčevMiklós TakóBojana VidovicMikołaj GralakBo-Kang LiouMilad HadidiBoli GuoMilan HouškaBomi RyuMilan Kumar LalBonggi LeeMilan MarkovicBon-Jae GuMilan MirosavljevićBonnie CanzianiMilan SkalickýBoredi ChidiMilena MatejovičováBoredi Silas ChidiMilena PetriccioneBoris AntunovićMilena RašetaBoris BasileMilena TzanovaBoris KaretkinMilena VasicBoris U. StambukMilena VukicBostan NazishMilica AcimovicBożena TyliszczakMilica MirkovićBrahim BchirMilica NicetinBranimir PavlićMilica PopovicBranimir UrlićMiljana DjordjevićBranislav ŠojićMiljenko KonjačićBranka LevajMilka MilevaBrankica KartalovićMilna Tudor KalitBranko PopovićMiloš RadosavljevićBratko FilipičMilton De Jesus FilhoBrenda A. Silva-EspinozaMilva PepiBrendan NiemiraMin HuangBrett J. SavaryMin ParkBrienna L. Anderson-CoughlinMina KafkaletouBrij Pal SinghMinaxi SharmaBruce. D. HammockMindaugas LiaudanskasBrugère HubertMingchih FangBruna CarbasMingjing ZhengBruna De FalcoMing-Ju ChenBruna MattioniMing-Kuei ShihBrunella PerfettoMingzhou ZhangBruno Gonçalves BotelhoMinh HaBuddhika MadurapperumaMinhaz AhmedBuket Er DemirhanMin-Pei LingBukola G. OlutolaMioara BercuBulent BasyigitMiona BelovićBurak DemirhanMiranda SertićBurcu Öztürk-KerimoğluMirari ArancibiaC. P. A. Van WagenbergMircea Adrian OroianCaleb NindoMircea VinatoruCalogero DibellaMirco VaccaCamelia Elena LuchianMire ZlohCamelia F. OroianMirela AhmadiCamelia VizireanuMirela Baus LončarCamilla CattaneoMirela IličićCamille DugardinMirela Ivančić ŠantekCamilly FratelliMirela KopjarCarla BritesMirella De BellisCarla GentileMiriam ApelCarlo CamerlingoMiriam Ortega-HerasCarlo CatassiMiriam SelaniCarlo CosentinoMiriam TufarielloCarlo Giuseppe RizzelloMirian PateiroCarlo MorelliMiriana DuranteCarlo ZamboninMirja AhmmedCarlos A. Ledesma EscobarMirja Kaizer AhmmedCarlos Alfonso Álvarez-GonzálezMirjana B. PešićCarlos ÁlvarezMirjana D. MarčetićCarlos AydilloMirjana MihailovićCarlos Dias PereiraMirjana R. DimitrijevicCarlos Iván Delgado-NieblasMirjana RadomirovicCarlos Jiménez-CorteganaMirjana Rajilić-StojanovićCarlos Leonardo Céspedes AcuñaMirjana TurkaljCarlos Manuel SilvaMirko PeranoCarlos Pasqualin CavalheiroMirna Fuka MrkonjičCarlos Regalado-GonzálezMirna Leonor Suárez-QuirozCarlos Sandoval-CastroMiroslav FišeraCarlos SanzMiroslav HadnađevCarlos VasconcelosMiroslav JůzlCarlos Zuluaga-DomínguezMiroslav M. NovakovićCarlotta GirominiMirosław AniołCarlotta SavioMirosław GiurgCarmela LamacchiaMirosław MaziejukCarmela M. A. BaroneMirosława ChwilCarmela TripaldiMirosława Karpińska-TymoszczykCarmen Avilés RamírezMirsada SalihovicCarmen CuadradoMirta CrovettoCarmen Del Toro SánchezMitja KrizmanCarmen Eugenia SîrbuMito KokawaCarmen J. Contreras-CastilloMladen BrnčićCarmen Licon-CanoMoawiya HaddadCarmen M. Breitling-UtzmannMoazzam Rafiq KhanCarmen M. Cabrera MoralesMohamad Mazen Hamoud-AghaCarmen RaclesMohamed A. A. AhmedCarmen RubioMohamed A. HassanCarmen Sílvia Fávaro TrindadeMohamed AddiCarmine FinelliMohamed Al-FatimiCarmo SerranoMohamed Aymen ChaouchCarola CappaMohamed BazaCarolina Chávez-MurilloMohamed BouhrimCarolina FloresMohamed DahmaneCarolina FredesMohamed FaragCarolina Muñoz-GonzálezMohamed Fawzy RamadanCaroline MoraesMohamed FodaCarsten Fauhl-HassekMohamed G. ShehataCarsten KrischekMohamed Hussein Hamdy RobyCasarotti Sabrina NevesMohamed Mahmoud DeabesCasimiro MantellMohamed MohanyCatarina SeabraMohamed S. NafieCatarina SilvaMohamed Said AbbasCaterina RufoMohamed ZommitiCatherine LacosteMohammad Abdus SalamCatherine M. McauleyMohammad Afzal HossainCatrin TylMohammad Ali HesarinejadCayetano Medina-MolinaMohammad Ali Jabraeil JamaliCecil RannouMohammad AltamimiCecilia GeorgescuMohammad Bagher HassanpouraghdamCeferino Carrera FernándezMohammad FikryCélia C. G. SilvaMohammad GoliCelia CarrilloMohammad HojjatiCem DirekoğluMohammad IkramCengiz GökbulutMohammad Jamshed SiddiquiCennet Pelin Boyaci GunduzMohammad MasukujjamanCésar C. Leyva-PorrasMohammad MohsenzadehCesar Leobardo Aguirre-MancillaMohammad Reza FathiCesar OzunaMohammad RostampourChagam Koteswara ReddyMohammad Shah JahanChaiyaso ThanongsakMohammad Zahirul IslamChaiyavat ChaiyasutMohammadian MehdiChampa WijekoonMohammadreza RezaeigolestaniChandra Sekar Chandra MohanMohammed BuleChandran MasiMohammed El-MagdChandran SomasundramMohammed Fouad El BasuiniChang ChenMohammed GagaouaChang Joo LeeMohammed IddirChangling HuMohammed Rizwan KhanChangqi LiuMohd AdilChangwei HsiehMohd AdnanChang-Wei HsiehMohd Ezuan Bin KhayatChang-Won ChoMohd Hafis YuswanChanporn ChaosapMohd Nazam AnsariChantelle HumanMohd Nazri IsmailChanthima PhungamngoenMohd Razif ShahrilChao GuMohd SaeedChao Kai ChangMohd Salahuddin Mohd BasriChao KangMohit TyagiChao-Hui FengMohsen AkbarianCharalambos FotakisMohsen GavahianCharalambos KanakisMojca KorošecCharalampos KarantonisMojtaba YeganehCharalampos ProestosMolla MengistCharin TechapunMona IsmailCharlene B. Van BuitenMónica CarreraCharles BrennanMónica CostaCharles CrapoMónica Graciela ParisiCharles Odilichukwu R. OkpalaMónica L. Chávez-GonzálezCharles R. FrihartMónica Martínez-BlancoCharu GuptaMonica R. NemţanuChatchawal WongchoosukMonica RagusaChau Nguyen DinhMonica Rosa LoizzoChen TanMonica TarceaCheng Ee MengMonica TrifCheng LiMónica VieiraCheng LiuMonideepa Bhattacharya BecerraChengli HouMonika BorkowskaChengyu GaoMonika CzerwinskaChengzhen LiuMonika GarbowskaChia-Min LinMonika GargChiara ConchioneMonika JanowiczChiara Di LorenzoMonika KaraśChiara La TorreMonika KosmalaChiara MagniMonika Łopuszańska-DawidChiara Roberta GirelliMonika Marcinkowska-LesiakChieh-Yu LinMonika Mieszczakowska-FrącChien Wei OoiMonika Osińska-JaroszukChien-Hsun HuangMonika PischetsriederChien-Hung LeeMonika RomanChih Yuan KoMónika Stéger-MátéChih-Chung WuMonika SujkaChih-Wei ChangMonika ThakurChikkaballpur Krishnappa SunilMonika TrzaskowskaChlebowska-Śmigiel AnnaMónika ValdenegroChongwen WangMonika WereńskaChris DouvrisMonika WójcikChris HamiltonMonika ZiomekChrissie ButtsMonique GermoneChrissoula VoidarouMonjurul HaqChristelle Bou-MitriMontserrat DuenasChristelle GuilletMontserrat Martínez-PinedaChristian AndrighettoMontserrat MestresChristian Campos-JaraMontserrat Riu-AumatellChristian JungnickelMoon-Suhn RyuChristian MagniMorena GabrieleChristian PhilippMorteza AkbariChristian R. Encina-ZeladaMorteza KhomeiriChristian RothMoslehpour MassoudChristian SallesMostafa AhmadvandChristiane HilgerMostafa ElachouriChristianne Elisabete Da Costa RodriguesMostafa El-SheekhChristina C. TamMostafa SoltaniChristina E. LarderMouhsine GalaiChristoph GerhardMouming ZhaoChristoph LüthiMousa M. AlreshidiChristoph MüllerMozaniel OliveiraChristopher BallmannMpho E. MashauChristopher P. F. MarinangeliMrinmoy GhoshChristopher P. MattisonMrutyunjay JenaChristopher TaylorMuawuz IjazChristos PappasMudasir RashidChristos VlachokostasMuddasarul HodaChrysanthi PankouMudjekeewis D. SantosChrysoula TassouMüge ŞahinChuanjun LiuMuhamad Hafiz Abd RahimChuleui JungMuhamad Shirwan Abdullah SaniChung Sun-WooMuhammad AfzaalChung-Jen HuangMuhammad AslamChun-Hui ZhangMuhammad Azam KhanChunqing AiMuhammad Bilal SadiqChun-Yao YangMuhammad Faisal M.Chun-Yung HuangMuhammad Faisal ManzoorChuping LeeMuhammad Hayat JaspalChutima LimmatvapiratMuhammad Hazwan HamzahChye F. Y.Muhammad ImranCindy Leonor Alves DiasMuhammad Imran TousifCinthia Baú Betim CazarinMuhammad Issa KhanCíntia Ferreira-PêgoMuhammad JunaidCinzia MannozziMuhammad Kashif Iqbal KhanClaire PulkerMuhammad KhanClaire Sulmont-RosséMuhammad MunirClara Asunción Tovar RodríguezMuhammad NadeemClara C. S. SousaMuhammad Nadeem HassanClara Cecília Santana SousaMuhammad NaeemClara CicatielloMuhammad Saad MemonClara FaresMuhammad ShahidClara GrossoMuhammad SohaibClara TalensMuhammad SohailClarice SteffensMuhammad Syafiq Hazwan RuslanClarisse NobreMuhammad UllahClaudia Brünen-NiewelerMuhammad UmairClaudia FoersterMuhammad Usman KhanClaudia Giovagnoli-VicuñaMuhammad Waheed IqbalClaudia Ibacache QuirogaMuhammad Waqar AlamClaudia KeilMuhammad ZareefClaudia LeoniMuhammed ArslanClaudia Maria BalzarettiMuhammed AtamanalpClaudia OchoaMuhammed DumanClaudia Yuritzi Figueroa-HernándezMuhammed KüpeClaudio AlvarezMuhammed SaeedClaudio CaprariMuhammed YuceerClaudio CassinoMuhammet Ali GundesliClaudio FerranteMuhammet Irfan AksuClaudio LombardelliMuhittin KulakClaudio MedanaMuhsan EhsanClaudio ParraMükerrem KayaClaudio Rios-VelascoMukesh SinghCledir SantosMunikumar ManneClemencia Chaves-LopezMurad Al-HolyClemens MostertMurali DamaClemens PeterbauerMurali S.Clemens Von SchackyMurugesan ChandrasekaranCleydson Breno R. SantosMusfirah ZulkurnainClive H. YenMusio BiagiaConcepció AmatMustafa MortasConcepción Pérez-LamelaMustafa Nadhim OwaidConcetta NazzaroMustafa OzCong KongMustafa TuzenConrad MurendoMustafa YamanConrad PereraMutamed AyyashConsolatina LiguoriMuthu DharmasenaConstantinos G. TsiafoulisMuthu ThiruvengadamConstantinos GeorgiouMuthusivaramapandian MuthurajConstanza CardenasMuttalip GündoğduCoombs CassiusMuzzamal HussainCorina Aurelia ZugravuMyriam AbboudCorinne Pau-RoblotMyriam Van WinckelCorliss Ann O'BryanMyung-Ji SeoCornelia MirceaNa GaoCornelia VasileNa ZhangCorrado RizziNabi ShariatifarCostanza CeccantiNabil ElsheeryCostas E. StathopoulosNacer BellalouiCourage DzahNada BenajibaCrina Carmen MureșanNada M. MostafaCristhian CarrascoNada NikolićCristi SpulbarNada SmigicCristian AranedaNadia BadolatiCristian BernardiNadiya BoykoCristian Jiménez MartínezNae-Cherng YangCristian MartinezNafees AhemadCristian MoisaNaga Kr GhattamaneniCristian Torres-LeónNagah ArafatCristiana NunesNagaia CiacciCristiane AguiarNagendra ChauhanCristiano Augusto BallusNahla A. AyoubCristiano D'AndreaNa-Hyung KimCristiano GarinoNaima G. Cortes-PerezCristina AlmeidaNaira SahakyanCristina Bianca PocolNajla AltwaijryCristina BotinesteanNakako KatsunoCristina CalejaNakphaichit MassalinCristina Cebrián-TarancónNalan GokogluCristina CruzNanasaheb. D. ThoratCristina DamianNancy JerezCristina Delgado-AndradeNanjing ZhaoCristina ForzatoNannan XiCristina González-DíazNantawan TherdthaiCristina M. SenaNaoufal El HachlafiCristina Medina-PlazaNaphaporn ChiewchanCristina MellinasNarae LeeCristina Mihaela NicolescuNarandalai DanshiitsoodolCristina MinnelliNarayanankutty ArunaksharanCristina Miranda GuedesNarayanasamy Marimuthu PrabhuCristina Perez-SantaescolasticaNaresh Kumar MehtaCristina PrietoNasir KhanCristina ProserpioNastia VasilevaCristina Raluca Gh. PopescuNatal’Ya L. VostrikovaCristina SgherriNatalia CampilloCristina SoaresNatalia DrabińskaCristina TodaşcǎNatalia GrubaCristina Tristão AndradeNatalia J.Cristina VergaraNatalia JatkowskaCsaba HanczNatalia Nowacka-JechalkeCsilla Mohácsi-FarkasNatalia Rosa-SibakovCsilla SzebenyiNatalia Wiktorczyk-KapischkeCui-Ping FengNatalija Ursulin-TrstenjakCulleré LauraNatalja Pustovalova NørskovCunshan ZhouNataša HojnikCyril ColasNataša HulakCyril HrnčarNatasa JokovicDaeung YuNataša LjubičićDag EkebergNataša MikulecDagmar SchoderNataša NastićDagmara Wróbel-BiedrawaNatasha LoganDagmara ZłotkowskaNathdanai HarnkarnsujaritDaisuke KobayashiNattakarn AwaiwanontDaisuke KyouiNattapol TangsuphoomDaiva LeskauskaitėNaveen MahantiDaiva NielsenNavid Jubaer AyonDalibor ŠatínskýNavin RastogiDaliborka Koceva KomlenićNawal DubeyDalija SeglinaNayan Ranjan SinghaDamian OnwudiweNazarena CelaDamião Pergentino De SousaNazia HossainDan C. VodnarNebojsa IlicDan Cristian VodnarNebojša NedićDan LiNeda AničićDana Maria MunteanNeda O. ĐorđevićDana MiddendorfNeđeljko KarabasilDanai BoonyakiatNeela SatheeshDandan ZhaoNeelesh Kumar MehraDanh VuNeelima MahatoDanhui WangNeerja Mittal GargĐani BenčićNeerja SharmaDanica ZarićNeetu Kumra TanejaDaniel A. ValleroNegin NooriDaniel Alejandro BarrioNeil FitzgeraldDaniel Fábio KawanoNeith PachecoDaniel González-HedströmNejc UmekDaniel GranatoNela Nedić TibanDaniel Jacobo-VelazquezNellie TsipouraDaniel López MaloNelly DatukishviliDaniel MatulićNelson Gutiérrez GuzmánDaniel OgundijoNelson MarmiroliDaniel RealNelson PéRez GuerraDaniel SimeanuNemanja TeslicDaniel VertedorNemat AliDaniel WillettNenad MagazinDaniel ŻmudzińskiNerdy NerdyDaniela Amidžić KlarićNerilson Marques LimaDaniela BordaNermeen YosriDaniela CampanielloNermina SpahoDaniela CovinoNeşe ÇınarDaniela F. OliveraNesrein M. HashemDaniela FreitasNesrin Ecem BayramDaniela GiannettoNestor Gutiérrez-MéndezDaniela GrulovaNetto Flavia MariaDaniela HanganuNevijo ZdolecDaniela Herrera-BalandranoNguyen Quang TrungDaniela IstratiNguyen T. NhungDaniela KarashanovaNguyen Van QuanDaniela Sánchez AldanaNgyen QuangDaniele CarulloNick W. SmithDaniele F. MaffeiNicki EngesethDaniele Maria-FerreiraNicola CaporasoDaniele NaviglioNicola CiceroDanielle GreenbergNicola CondelliDaniil N. BratashovNicolás Laguarda-MiróDanijel D. MilinčićNicolas SommererDanijel KarolyiNicolas Yanou NjintangDanijela AšpergerNicole ParsleyDanijela Bursac KovacevicNicole Roberta GiuggioliDanijela Horvatek TomicNicoleta RaduDanijela SkrozaNicoleta-Aurelia ChiraDanijela ŠuputNicoletta GuerrieriDanilo Escobar-AvelloNiculaua M.Daniso BeswaNiculaua MariusDanka BukvickiNidal JaradatDanny Lee RinkerNidhi AdlakhaDante J. BuenoNidhish FrancisDanuta JaworskaNidia Casas-ForeroDanuta Kołożyn-KrajewskaNiël Van WykDanyang YingNiels HansenDão Pedro De Carvalho NetoNifei WangDaohua XuNiina HaiminenDaozong XiaNika PavlovićDarija CörNikhil ChrungooDarinka AckovaNikita Khromov BorisovDario FrisoNikita TsvetovDario Iker Tellez-MedinaNikki ShariatDario KremerNikola ČobanovićDario LasićNikola MilicevicDario PaoloNikola PopovićDariusz GóralNikola TomicDariusz GozdowskiNikolaos KontoudakisDariusz M. StasiakNikolaos KopsahelisDarko PreinerNikolina Kelava UgarkovićDasha MihaylovaNilesh NirmalDavid BiedermannNilgün ÖzdemirDavid CastrilloNima HematyarDavid ChelazziNima SanadgolDavid I. WilsonNina Batorek LukačDavid Juárez VarónNina RecekDavid L. HopkinsNiny Z. RaoDavid LaureysNiramon Utama-AngDavid N. CoxNiranjan ParajuliDavid NuttNirmal MazumderDavid ObenlandNishant KumarDavid RanucciNishesh SharmaDavid S. HenriquesNitin DhowlagharDavid ŠilhaNitin MehtaDavid SykoraNitish KumarDavid Ward RoubikNives Marušić RadovčićDavide BarrecaNiya ZhangDavide BertelliNobuo SuzukiDavide GiacaloneNoé OntiverosDavide GottardiNoelia BetoretDavide SlaghenaufiNoelia Rosales-ConradoDavit PipoyanNoemí Echegaray SuárezDavood ZareNoha H. HabashyDavor ValingerNoman WalayatDa-Wei HuangNoorakmar Ab. WahabDayun YanNoorfatimah YahayaDe Carvalho Julio CesarNooshin SohaniDeana JonesNopakarn ChandetDebabrata SircarNor QhairulDebasish Kumar DeyNóra AdányiDeblina BiswasNóra BákonyiDebojyoti MoulickNorhashila HashimDébora CamposNorhasnida ZawawiDébora Cerdá-BernadNorizah Mhd SarbonDeepak J. SharmaNorlia MahrorDeepak VermaNorma Francenia Santos-SánchezDeepika SinghNoura DosokyDeisy Alessandra DrunklerNoureddine ElboughdiriDejan PrvulovićNoureddine IssaouiDejan StojkovićNourhéne Boudhrioua-MihoubiDele OgunremiNqobile MasondoDelphine VincentNugraha SuyatmaDemetris KafourisNuno Bartolomeu AlvarengaDequan ZhangNuno Miguel Fonseca FerreiraDespina KalogianniNunzia CiccoDevin RoseNur Alim BahmidDhaliwal Salwinder SinghNur CebiDhananjay YadavNurhan ErtașDharmendra YadavNurkhalida KamalDhiraj VyasNurul Aqilah Mohd ZainiDiana Andreia Tavares PintoNurul Hanisah JuhariDiana CholakovaNurul HudaDiana De SantisNuzhat HumaDiana DumitrasOana Viorela NistorDiana Hernandez-RomeroObaid AfzalDiana I. SantosOctavian Tudorel OlaruDiana Maria VranceanuOctavio Dublan-GarcíaDiana ObistioiuOctavio Elizalde SolisDiana Roxana PelinescuOctavio GarciaDicky Tri UtamaOctavio Paredes-LópezDidar UcuncuogluOgueri NwaiwuDidem AykasOguz BayraktarDidem Sözeri AtikØivind BerghDidier BrassardOksana SintsovaDidier StuergaOksana ZininaDiding SuhandyOla LasekanDiego A. CastellanosOlaf LarsenDiego A. MorenoOlaia Martínez GonzálezDiego Ballesteros-VivasOlcay Kaplan InceDiego BonattoOlesia MakhutovaDiego F. TiradoOlga A. KoksharovaDiego García-GomezOlga AmaralDiego La MendolaOlga B. Alvarez-PerezDiego MoralesOlga MitrovićDiego PlanetaOlga Monago-MarañaDiego TaladridOlga Rojo-PovedaDietmar EnkoOlga TsivilevaDilhun Keriman Arserim UcarOlimpio MonteroDima Faour-KlingbeilOliver MeixnerDimitra DimitrellouOliver TuševskiDimitra HouhoulaOlivera GalovićDimitrina Zheleva-DimitrovaOlivera KoprivnjakDimitrios A. AnagnostopoulosOlivia LapentaDimitrios GerasopoulosOlivier GibertDimitris ArapoglouOliviu VostinaruDimitris SkalkosOlja ŠovljanskiDina El-KershOlumide A. OdeyemiDina Helmy El-GhonemyOlusola Adedayo AdesinaDina LončarićOluwatoyin O. OnipeDinca Oana BotoranOm Prakash MalavDinesh KumarOmar Guzman-QuevedoDingbo LinOmobolanle (Bola) OloyedeDinithi PeirisOnay Burak DoganDinushani Anupama DaranagamaOndrej HanušovskýDiogo CunhaOng Meng ChuanDiogo Thimoteo Da CunhaOns BouchamiDiomi MammaOriana GavaDirk LachenmeierOriana MottaDirk TischlerOrtega-Hernández ErikaDirk W. LachenmeierOscar Alberto Alvarez SolanoDivyang SolankiOscar González-RíosDjenane DjamelOscar NunezDjordje MedarevicOsman ErkmenDmitriy SmolenskyOsmar Antonio Jaramillo-MoralesDo Yup LeeOsvaldo ResendeDoha A. MohamedOtilia BobisDoina Georgeta AndronoiuOtilia CintezaDolores González De LlanoOttmar DistlDolores R. SerranoOvidiu Cristian OpreaDomenico GabrieleOvidiu TitaDomenico MeloniOwen Griffith JonesDomenico MontesanoOya Irmak SahinDomenico Pietro Lo FiegoOzcan TulayDomenico SagnelliÖzgür KuzukıranDomingo FernándezOzgur TarhanDomingo Saura LópezOzlem Yesil-CeliktasDominic AugustineOzlem YilmazDominic NarangPablo E. HernándezDominico A. Guillén-SánchezPablo FaríasDominik KmiecikPablo MelgarejoDominik MierzwaPablo MirallesDominik ZimonPablo RibottaDominika AdamczykPablo SobradoDominika GlabskaPadma Ishwarya S.Dominika GuzekPajor FerencDominika Średnicka-ToberPalaniyandi KaruppaiyaDominika VešelényiováPaloma LopezDonatella BulonePamela ManziDonatella RestucciaPan WangDonato CalabriaPanagiota-Kyriaki RevelouDong Uk AhnPanagiotis KandylisDong XinPanagiotis KarayannakidisDongdong WangPanagiotis SimitzisDong-Min ShinPankaj PathareDora Allegra CarbonePanmanas SirisomboonDora MelucciPantelis NatskoulisDorina CasoniPantu Kumar RoyDorota Cais-SokolińskaPaola CuomoDorota DerewiakaPaola Di MatteoDorota GałkowskaPaola Gauffin-CanoDorota Grabek-LejkoPaola Hernández-CarranzaDorota Najgebauer-LejkoPaola RomecínDorota NowakPaola Sánchez-BravoDorota OlenderPaola SangiorgioDorota Piasecka–KwiatkowskaPaola TedeschiDorota SosnowskaPaolo BertoncelloDorota WianowskaPaolo ConflittiDorota WojniczPaolo ContiDorota ŻyżelewiczPaolo DaminelliDouglas BarbinPaolo D'InceccoDouglas ConstancePaolo GabrielliDragan AntićPaolo PolidoriDragan CvetkovićPapungkorn SangsawadDragan MilicevicParaskevi MitliagkaDragan ŽikićPardeep Kumar SadhDragan ŽnidarčičParise AdadiDragana BožićParizad Parisa AbbasiDragana DabićParmpal S. GillDragana Ljubojević PelićParthasarathi SubramanianDragana Šoronja‐SimovićParthasarathy SrinivasanDraghici OlgaPascal GerbauxDrago BesloPasquale CatalanoDrago KočarPasquale CrupiDragoljub CvetkovićPasquale GiungatoDragoljub GajicPasquale Massimiliano FalconeDražen LušićPathima UdompijitkulDrew BudnerPatricia CarloniDriss OusaaidPatrícia MartišováDuangjai TungmunnithumPatricia Reboredo-RodríguezDuarte Correa Yudy StellaPatrícia ValderramaDubravka NovotniPatricio Orellana-PalmaDubravka ŠkrobotPatricio PalmaDubravka Vitali ČepoPatrick PoucheretDunhua LiuPatrick VanraesDunja ŠamecPatrizia FavaDuqin ZhangPatroklos VareltzisĐurđica AčkarPaucean AdrianaDuried AlwazeerPaul Baruk Zamudio-FloresDurmuş SertPaul GardDušanka A. PopovićPaul KilmartinDuygu AğagündüzPaul NkegbeE. F. Simó-AlfonsoPaula A. OliveiraE. L. PereirPaula AlvitoE. Radziejewska-KubzdelaPaula BorrajoE. V. SchmalhausenPaula C. B. PaígaE. Z. PanagouPaula C. CastilhoEbrahiema ArendsePaula CorreiaEbrahim TaghinezhadPaula GarcíaEbru KafkasPaula J. JauregiEd G. M. Van KlinkPaula JimenezEdgar CambazaPaula PereiraEdgar Tavares-da-SilvaPaul-Alexandru PopescuEdgar Torres-MaravillaPaulina BierzuńskaEdgardo GiordaniPaulina KęskaEdith Ponce-AlquiciraPaulina Pogorzelska-PrzybyłekEdson SilvaPauline OngEduard-Marius LungulescuPaulo CardosoEduardo Augusto Fernandes NilsonPaulo De Souza Costa SobrinhoEduardo Cesar TondoPaulo Eduardo MunekataEduardo CostaPaulo ValeEduardo EstevesPavan K.Eduardo Festozo VicentePavan KumarEduardo Fuentes-LemusPavel DostalekEduardo MoralesPavel MokrejšEduardo Morales-SánchezPavel NevrklaEduardo Yoshio NakanoPavla LakdawalaEdward FuentesPavol FarkašEdward MajewskiPaweena PongdontriEdward MunteanPawel BrylaEdward RojPaweł GlibowskiEdy Sousa BritoPaweł HanusEdyta Gendaszewska-DarmachPawel KafarskiEdyta Kowalczuk-VasilevPaweł KonieczkaEdyta SymoniukPawel KonieczynskiEero PuolannePaweł LenartowiczEffarizah Mohd EsahPaweł SatoraEfigenia Montalvo GonzálezPaweł ŚwitEfrain Manuelito Castro AlayoPaweł ZagrodzkiEgan DoevenPawin PadungtodEgeria ScodittiPeano CristianaEgon Henrique HorstPedro Aguilar-ZarateEgor OsidakPedro AraujoEhsan AhmadpourPedro BrancoEhsan Moghaddas KiaPedro Cerezal-MezquitaEirini PanagopoulouPedro De Magalhaes PadilhaEishin MoritaPedro FernandesEkaterina A. LitvinovaPedro Ferreira-SantosEkaterina A. ZelentsovaPedro González-RedondoEkaterini MoschopoulouPedro N. S. SampaioEl Hadi ErbiaiPedro Rodríguez-LópezEl Hassan SakarPedro SantosElahe AbediPedro SilvaElaine MeloPedro SouzaElena BozzettaPedro Ulises Bautista-RosalesElena Coyago-CruzPeeyush SoniElena Falqué LópezPeihua MaElena FranciosiPekka UimariElena GalassiPengfei HanElena KotenkovaPenghao WangElena KozhevnikovaPérez-Won MarioElena MakarovaPeriyanaina KesikaElena MolinaPeriyar Selvam SellamuthuElena NiccolaiPetar RistivojevićElena Sánchez-ZapataPeter CraggElena TorrieriPéter FodorElena VelickovaPeter HaščíkElena ViganòPeter J. MaughanElena VillacrésPeter J. T. DekkerElena-Daniela GrigorescuPeter MansellElena-Suzana Biriș-DorhoiPeter MurianaEleni BozinouPeter PaulsenEleni KakouriPeter TorleyEleni KasapidouPeter WankeEleni MalissiovaPetjon BallcoEleni ZafeiriouPetko AlovElenilson De Godoy Alves FilhoPetko DenevEleonora CariniPetra FörstEleonora MazzuccoPetra KotnikEleonora PagnottaPetra MatićElham AnsarifarPetra TurčićElham AzarpazhoohPetros KatapodisElham SarmastPhebe DingElia RanzatoPhilip CrandallEliana Badiale FurlongPhilip HublitzEliana Marcela Velez-ErazoPhilip JakemanEliane CollaPhiliswa Nosizo NomngongoElif YildizPhillip E. StrydomElisa AlePhuong-Thao TranElisa De ArcangelisPichan PrabhasankarElisa Ferreira MouraPierangelo FreschiElisa GiampietriPierluigi RevegliaElisa Miriam Valenzuela SotoPiero FranceschiElisa RobottiPierre RougeElisabet Fernández-GómezPilar Colás-MedàElisabeta BotezPilar FrancinoElisabetta BonerbaPilar NavarroElisabetta MurruPilar ZafrillaElisabetta SchievanoPing DongElisete MourãoPing XiangEliza KostyraPingkeng WuElizabeth Carvajal-MillanPiotr BąskaElizabeth TomasinoPiotr BórawskiElizabeth TroncosoPiotr HapetaElizabeth TymczyszynPiotr HeczkoElke AlbrechtPiotr IwaniukEllen Gelpi-MantiusPiotr JaniszewskiEllen HertzmarkPiotr KapustaElnaz ZeynalooPiotr KubiakEloisa Dutra CaldasPiotr KuśElsa Díaz-MontesPiotr KuźniarElsa F. VieiraPiotr LatochaElsa Margarida GonçalvesPiotr MichelElsa MechaPiotr SkałeckiElsa RamalhosaPiotr ŚwiątekElsa UribePiotr SzwedaElsa VascoPiotr ZarzyckiElsayed E. HafezPiya TemviriyanukulElsayed MehanaPiyaluk NurerkElsje PietersePo-Hsien LiElvyra JarienėPoliana Cristina SpricigoElżbieta Goryńska-GoldmannPomi ShahbazElżbieta Hać-SzymańczukPonnuswamy VijayaraghavanElżbieta KarlińskaPooja PandeyElzbieta KlewickaPoonam MishraElżbieta Łopieńska-BiernatPoonam SharmaElzbieta RosiakPopa Stefan-LucianElżbieta SuchowilskaPorfirio Gutierrez MartinezEmad Al-DujailiPornthap ThanonkeoEmad ShalabyPouria AtaeiEman abd Elmenam Ahmed MahmoudPo-Wen ChenEman E. AbdeenPo-Yuan ChiangEman M. OthmanPrabhu PonnandyEmanuel PeresPrabin LamichhaneEmanuela CamilliPrabir K. SarkarEmanuela PanniaPrachurjya DuttaEmanuela SalviatiPradeep DwivediEmanuela TrovatoPradeep Kumar PandaEmanuela ZanardiPradip BehareEmanuele RizzoPradyuman KumarEmel OzPrakash K. NayakEmilia AlvesPramod K. PrabhakarEmilia DrozłowskaPranas ViškelisEmilia FornalPranav ChauhanEmilia Garcia-MorunoPranav Kumar PrabhakarEmilia Janiszewska-TurakPrasad ChavanEmilia PapakonstantinouPrasad RasaneEmilia VassilopoulouPrasanna BhaleraoEmilio Álvarez-ParrillaPrasanna GunathilakeEmilio V. Carral VilariñoPrasanna RamaniEmin YılmazPrasanna VaddiEmmanouil D. TsochatzisPrasanna VasuEmmanouil KokkinakisPraseptiangga DanarEmmanouil PapaioannouPrathap SomuEmmanouil TsochatzisPredrag IkonićEmmanuel NjogaPredrag PetrovićEmmanuel PetitPreetha Preetha RadhakrishnanEmmanuela MagriplisPreeti ChandraEncarna Gomez PlazaPrince ChawlaEndler BorgesPriscilla AmaralEndre MathePriscilla Moura RolimEng Shi OngPriyankar DeyEng-Keng SeowProdromos SkenderidisEnric I. CanelaProkopios MagiatisEnrico DoriaProspero Di PierroEnrico GrecoPrzemysław KosobuckiEnrico Karsten HaddePrzemysław KowalczewskiEnrico Maria MosconiPrzemysŁaw ł. ZalewskiEnrico NovelliPrzemyslaw SiejakEnrico Vito PerrinoPui Liew PhingEnrique Domínguez ÁlvarezPulatsu EzgiEnrique MejíasPurificación García-SegoviaEnza D'AuriaPutri HarlinaEnzo SpisniQamar ZamanEr Sheng GongQi WangEric A. DeckerQian ChenEric Keven SilvaQian ZhangÉrica S. SiguemotoQiang FuErica VareseQiang LiuErich LeitnerQiang YuErick César López-VidañaQiangzhong ZhaoErick Paul Gutiérrez-GrijalvaQianwen ZhangErick Sierra-CamposQing JinÉrico Chagas CaperutoQingli DongErik M. PilgrimQingsen ShangErika BujnaQiu-Jin ZhuErnesto A. Lagarda-LeyvaQiuqin ZhangErnesto Tarragon CrosQun CaoErzsébet SándorQuoc Cuong NguyenEsmaeil AbdollhazadehRachel GillespieEspérance DebsRachma WikandariEsra CapanogluRaciel Javier Estrada-LeónEşref DemirRadivoj ProdanovićEssam Mohamed ElsebaieRadmila PavlovicEstéfani García-RíosRadosław GwardaEstefanía Valero-CasesRadosław KowalskiEster TriguerosRadosław MącikEsther F. MyersRadosław SpychajEsther Gomez MejiaRadu C. RacovitaEsther Ramírez-MorenoRafael Apolinar-ValienteEszter NemeskériRafael DefendiEtna Aída Peña-RamosRafael G. Campos-MontielEugenia PapadakiRafael GavaraEulalia SkawińskaRafael Guillén-BejaranoEunice ValdugaRafael Paseiro-CerratoEva DudrikováRafaelle MarroneEva Garcia VazquezRafał NadulskiEva K. PressmanRafał OgórekEva M. Santos LópezRafał WawrzyniakEva Maria Navarrete-MunozRafał WołosiakEva NavascuesRafał ZiobroEva PinhoRaffaele ZanoliEva SamkováRaffaella BranciariÉva SzabóRaffaella ColomboEva Tejedor-CalvoRaghavan VijayaEva TsaousidouRahul MangayilEva WiklundRainer BussmannEva Zamborine-NemethRajesh Kumar VishwakarmaEvagelos GikasRajesh WaghEvandro Leite De SouzaRajiani IsmiEvangelia FappaRajinder PalEvangelia Litsa TsianiRajiv DahiyaEvangelina García-ArmentaRajka BozanicEvasio PasiniRalf BlankEvelyn MadorobaRalf G. BergerEvita StraumiteRaluca Șuică-BunghezEwa Czarniecka-SkubinaRaluca-Giorgiana ChivuEwa DłuskaRam PrasadEwa DomianRamachandran IshwaryaEwa GondekRamalingam AnantharajEwa HalickaRamesh Kumar SainiEwa Jabłońska-RyśRamesh NagarajappaEwa JakubczykRameshthangam PalanivelEwa LangeRamiro Baeza-JiménezEwa MajewskaRamiz AlievEwa Ostrowska-LigęzaRamon Canela-GarayoaEwa RopelewskaRamon JaimeEwa SzpyrkaRamon RochaEwa ZdybelRamsés Ramón González EstradaEwelina HallmannRana Muhammad AadilEwelina KowalczykRana MustafaEwelina PatyraRando TuvikeneEwelina Pogorzelska-NowickaRandy AdjonuEwelina WęsierskaRanko RomanićEwelina ZielińskaRaphaëlle SavoireEwen ToddRaquel Braz Assuncao BotelhoFabián GuillénRaquel MoralFabiana Perrechil BonsantoRaquel P. F. GuinéFabiane BachRaquel SoengasFabiano André Narciso FernandesRaquel TorrijosFabio BozzoliRasa ZukieneFabio BrambillaRashid A. N. M. MamunFábio De Souza DiasRathinam RajaFabio Di NardoRatmawati MalakaFabio FanariRaúl Alberto Reyes-VillagranaFabio FavatiRaul DomínguezFabio Gaetano SanteramoRaul GirardelloFabio Granados-ChinchillaRaúl Pérez-GálvezFabio MencarelliRavi Kant AgrawalFabio MussoRavi ManiFabio PaceRavi PandiselvamFabio SciubbaRavinder KaushikFabio VerneauRavindra Pratap SinghFabiola Castorena-TorresRaviraj ShindeFabrizia TittarelliRavish ChoudharyFabrizio AnniballiRay GlahnFabrizio CincottaRaymond J. OwensFadia YoussefRay-Yu YangFady MoharebRaziye PourdarbaniFakhri ShahidiRazvan DinaFan ZhaoRazzagh MahmoudiFang WeiRebeca Monroy TorresFangyu LongRebecca C. DeedFanny TsironiRebeka Kovacic LukmanFanta Desissa GutemaReema TayyemFarah Saleena TaipRegina Ngozi UgbajaFariborz HabibiRegina WierzejskaFarid ChematReham HamidaFarid SroorReham Hassan MekkyFarouq Heidar BaridoReham M. El-TarabiliFarrokh GhahremaninejadRei SonobeFarzad KianiRemedios YáñezFatehi FoadRemigiusz BąchorFatemeh GhiasiRenan FalcioniFatemeh Mohammadi-NasrabadiRenata BarrosFathiya KhamisRenata Cegielska-RadziejewskaFatih ÖzRenata NassuFatih OzogulRenata Puppin ZandonadiFatih TörnükRenata Pyz-ŁukasikFatiha BrahmiRénato FroidevauxFatima BentoRenato L. BinatiFátima De Cássia Oliveira GomesRenato S. CarreiraFatma BoukidRenato ZanellaFa-Wen YinRené R. Balandrán-QunitanaFederica BonelloRenpeng DuFederica CastellaniRey David Vargas-SánchezFederica Di CesareRiadh BadraouiFederica FinettiRiadh IlahyFederica IanniRiaz UllahFederica MurmuraRicard BoquéFederica PasiniRicard BouFederica RivaRicardo Cordero OteroFederica TaddeiRicardo González-RezaFederica TonoloRicardo Reyes-DíazFederica TurriniRiccardo AigottiFederico CasanovaRiccardo GuidettiFederico Maria RubinoRicha SalwanFeifei TaoRichard JägerFelice PanebiancoRichardos Nikolaos SalekFelicia ChețanRiddhiman DharFelipe Rodrigues Dos SantosRie YatsunamiFelix ClanchyRifat Ullah KhanFelix ZulhendriRiikka PeltomaaFeng LiRika Indri AstutiFengchao CuiRita CelanoFeng-Jie CuiRivka CahanFengzheng GaoRobert AsieduFerenc PelesRobert KunstFerhat YükselRobert NowackiFernanda AchimónRobert RusinekFernanda CosmeRobert T. MeansFernanda DiasRobert TylerFernanda Furlan Gonçalves DiasRoberta BattistiniFernanda Maria VaninRoberta ComunianFernanda OduberRoberta D’AurelioFernanda Papa SpadaRoberta EspositoFernanda SantosRoberta FoligniFernanda VilarinhoRoberta MoruzzoFernando Cordeiro RaposoRoberta NugnesFernando José PereiraRoberta PassafiumeFernando Jose SolanillaRoberta PreteFernando NorambuenaRoberta ThysFernando Octávio Queirós DouradoRoberta TolveFernando PablosRoberta ZupoFernando RamalhoRoberto AneddaFernando Ramos-EscuderoRoberto Castro-MuñozFerruccio GiamettaRoberto CiccorittiFestus BekunRoberto ConsonniFevzi BardakciRoberto Di MarcoFilip ŠupljikaRoberto GottiFilipa Rego PintoRoberto J. C. FonsecaFilipa Sofia Dinis ReisRoberto LemusFilipa Vinagre Marques SilvaRoberto MandrioliFilippo GiarratanaRoberto PisanoFilippo RossiRoberto StellaFilomena NazzaroRobin JoshiFinazzi GuidoRocky J. DwyerFiorella SinesioRodica Maria SimaFioretta AsaroRodica PamfilieFirdos Alam KhanRodolfo MascheroniFlavio BocciaRodolfo Ramos GonzálezFlora VitalisRodrigo A. IbáñezFlore DepeintRodrigo Fortunato De OliveiraFlorencia VersinoRodrigo GilFlorentina GateaRodrigo NovaFlorentina Laura ChiriacRodrigo Romero-NavaFlorentina Monica RadulyRodrigo StephaniFlorentina-Daniela MunteanuRodrigo TorresFlorin CiolacuRodrigo Wladimir ValenzuelaFlorin OanceaRodrigues Chaves AndreaFlorina DrancaRogelio Flores-RamírezFlorin-Dumitru PetrariuRogelio Sánchez-VegaFloris WardenaarRogério MendesFodor MariettaRoksana MarkiewiczFohad HusainRoman KotlowskiFoteini F. ParlapaniRoman PaduchFoteini PavliRomeo RojasFotios ChatzitheodoridisRommy ZunigaFotis TsopelasRomuald ZalewskiFouad AbdullahRonald Huarachi OliveraFouad Ali Abdullah AbdullahRonghai HeFouad JawabRongjin SunFouad Mahmoud Fouad ElshaghabeeRonit MandalFranca FinocchiaroRosa Colucci CanteFrancesc Fusté-FornéRosa Herráez-HernándezFrancesc SepulcreRosa Luisa AmbrosioFrancesca AielloRosa Maria FanelliFrancesca BiandolinoRosa María Mariscal MorenoFrancesca BlasiRosa RomeoFrancesca ConteRosa Wanda Diez-GarciaFrancesca De FalcoRosalía López-RuizFrancesca LionettoRosalva Mora-EscobedoFrancesca MalvanoRosana ChirinosFrancesca MartucciRosana RibićFrancesca MasinoRosanna Di PaolaFrancesca MeliniRosanna PaolinoFrancesca NocenteRosanna TofaloFrancesca PedoneseRosaria CozzolinoFrancesca ValerioRosário AnjosFrancesca VenturiRosario RamirezFrancesco BarrecaRosemary Aparecida De CarvalhoFrancesco BimboRosendo Balois-MoralesFrancesco BozzoRoshanida A. RahmanFrancesco CacciolaRosiane Aparecida MirandaFrancesco CaponioRosilda Mara MussuryFrancesco CaridiRosina López-FandiñoFrancesco CrudoRossana RoilaFrancesco DesogusRossella Di MonacoFrancesco GaiRossella SvigeljFrancesco GenoveseRothman KamFrancesco GeobaldoRovia KobunFrancesco GiannicoRoxana MedinaFrancesco MantaRoya R. R. SardariFrancesco Maria CalabreseRóża Biegańska-MarecikFrancesco MilanoRubén AgregánFrancesco PennisiRuben BustosFrancesco PomilioRubén Francisco González-LaredoFrancesco RuotoloRuchir PriyadarshiFrancesco SavoraniRudiati Evi MasithohFrancesco SerrapicaRuengwit SawangkeawFrancesco TiniRui CostaFrancesc-Xavier MedinaRui FaustoFrancis ButlerRui M. S. CruzFrancis MuchaambaRui Min Vivian GohFrancisc DulfRui PoinhosFrancisca RodriguesRuifen ZhangFrancisco C. IbañezRuilong ShengFrancisco Humberto Xavier-JuniorRung-Yi LaiFrancisco J. Aguirre-CrespoRuojun MuFrancisco J. BarbaRusen Metin YildirimFrancisco J. Salafranca LázaroRushdan A. IlyasFrancisco Javier Alvarez-MartinezRuslan MariychukFrancisco Jose Sarabia SanchezRussell J. MerrittFrancisco LesRuta GaloburdaFrancisco LopezRuta GruskieneFrancisco MendezRuzaina IshakFrancois SabotRyan ArdoinFrançoise DenisRyota HosomiFrank Blanco-PerezRyszard AmarowiczFrank PeelmanRyszard CierpiszewskiFrank SchurenRyszard RezlerFrank WelleRyszard SwietlikFrans Van KnapenSaad AlghamdiFransiscus SuryadiSabina GalusFrantišek BuňkaSabina Lachowicz-WiśniewskaFrantišek KrepsSabina PurkrtováFrantišek LorencSabira SultanaFred GatesSabna KottaFrederick SarpongSabreen Ezzat FadlFrederico BarrosSabuj Kanti MajumderFukun GuiSachiko KoyamaFunda Karbancioglu-GulerSachin SonawaneFurkan Türker SarıcaoğluSaddam SaqibG. Calderón-DomínguezSaeid HosseinzadehGabriel Abraham Cardoso UgarteSaeid JafariGabriel Aguirre-ÁlvarezSagar MaitraGabriel López-GarcíaSaid GharbyGabriel MustateaSaiful Irwan ZubairiGabriela AlbuquerqueSaikat MitraGabriela ChiciudeanSajad Ahmad SofiGabriela CristeaSajed AmjadiGabriela GrigioniSajid AliGabriela Inés De NoyaSajjalavarahalli G. Eswara ReddyGabriela Mariana Rodriguez-SerranoSakai KiyotaGabriela Medina-PérezSakamon DevahastinGabriela RapeanuSalam IbrahimGabriela Vollet MarsonSaleh Al-GhamdiGabriele DomenicoSalih KarasuGabrieli BernardiSalma Mohamad YusopGabriella GiovanelliSalvador E. Maestre PérezGabriella KiskóSalvador MáñezGabriella SavianoSalvatore BarrecaGabriella SiestoSalvatore VelottoGabriella TamasiSam Al-DalaliGabrielle GautérioSamad EbrahimiGabrijela Tavcar KalcherSamah El-SayedGaetano CammilleriSamala NagarajGaia MeoniSamart Dorn-InGali NavehSameh A. KormaGalina NikolovaSamer MudalalGamal MohamedSami Abou FayssalGamal Mohamed HamadSami GhnimiGamze YazarSami I. AliGanesan NarsimhanSamir DasGarry OsthoffSamir KalitGary D. RaysonSamir Mohammed Abd-ElghanyGary DykesSamo Mahnič-KalamizaGary GaoSamo SmrkeGary ThompsonSampat GhoshGaston Gutierrez GamboaSamuel Durán-AgüeroGastón TorrescanoSana AlibiGaurav Kr DeshwalSandeep GhatakGauri JairathSandeep JagtapGaurikutty PadmajaSandeep K. PandaGavino SannaSandeep K. SharmaGediminas NiauraSandeep KumarGeir MathiesenSandeep PandaGeir Sogn-GrundvagSandip GhugeGemma MoragaSandip M. VibhuteGemma Oms-OliuSándor BeszédesGengjun ChenSándor GondaGeni Rodrigues SampaioSandra BalbinoGeoff KiraSandra BorgesGeoffrey MuellerSandra CastilloGeok Lin KhorSandra ClarkeGeorge AbongSandra González-PalaciosGeorge AnnorSandra HillGeorge BotsarisSandra L. Gomez-PerezGeorge DaubeSandra Olarte-MantillaGeorge FthenakisSandra OsésGeorge KatsarosSandra Sofia Quinteiro RodriguesGeorge Mihail VlăsceanuSandra Sumalla-CanoGeorge SchollSandra ZavadlavGeorge SuciuSang Gil LeeGeorge ZachariadisSangbin LimGeorgi BeevSang-Han LeeGeorgia MandilaraSang-Hoon LeeGeorgia-Eirini DeligiannidouSangita GangulyGeorgiana CodinaSangmi LeeGeorgiana DeakSanil S. HishanGeorgiana Gabriela CodinăSanja Kostadinović VeličkovskaGeorgios BokiasSanja RadmanGeorgios DelisSanju Bala DhullGeorgios DiamantopoulosSanjukta SubudhiGeorgios TsaniklidisSanna ManuelaGeorgios XanthopoulosSantiago BenitoGerard DumancasSantiago Hurtado-BermúdezGerardo Fernández BarberoSantiago P. AubourgGerardo Mendez-ZamoraSantosh K. YadavGercino Ferreira Virgínio JúniorSantosh KumarGergő KallóSaqib ArifGergő TóthSaqib FarooqGerold BesserSara Alonso-TorreGerui RenSara CuellarGeun-Pyo HongSara ErasmusGhadha Ibrahim FouadSara HedayatiGhazala NawazSara PrimavillaGholamreza GohariSara R JaegerGholamreza Nikbakht BrujeniSara SaviniGhoson DabaSara SilvaGiacomo PetrettoSarah AzinheiroGiacomo SqueoSarana SommanoGiacomo ZaraSaranya PoapolathepGiampaolo ColavitaSarina Abdul Halim LimGiampiero PagliucaSarma MutturiGianfranco PannellaSaša DespotovićGianfranco PiconeSaša PrđunGianluca RizzoSascha RohnGianluigi CardinaliSatheesh NeelaGianluigi MaurielloSathiyaseelan AnbazhaganGianna FerrettiSatish KumarGianni ZoccatelliSaulo Luz SilvaGiannis TsoulfasSaurabh SinghGianpiero PataroSayra N Serrano-SandovalGilberto Vinícius De Melo PereiraScott M. HoltGilda AielloSebahattin TurgutGina AmbrosioSebastian BalickiGinny LaneSebastián EscobarGiordano RuggeriSebastian GranicaGiorgia LiguoriSebastiana FaillaGiorgia MoschettiŞebnem HarsaGiorgia PerpetuiniSebnem Ozturkoglu-BudakGiorgio SmaldoneSebnem TavmanGiovani Carlo Veríssimo Da CostaSeda ŞengülGiovanna BaronSeddik KhalloufiGiovanna BoschinSefater GbashiGiovanna CaparelloSehrish MananGiovanna EspositoSeiichi MatsugoGiovanna Rossi-MarquezSeinen ChowGiovanni AntoniniSeley GharaneiGiovanni BartolomeoSelim EsenGiovanni De FrancescoSelma Dry BerkGiovanni PeiraSelma Soares De OliveiraGiovanni PreitiSemaghiul BirghilaGiovanni SogariSencer BuzrulGiraudo AlessandroSendhil RamadasGisandro Reis CarvalhoSenem KamilogluGisèle LapointeSenem Kamiloglu BestepeGi-Seong MoonSenka DjakovićGitana AlenčikienėSenka PopovićGiulia AbateSeok Shin TanGiulia BianchiSeon-Min OhGiuliana DonadioSerena BandiniGiuliana VinciSerena MandolesiGiuseppe BlaiottaSergey MaksimovGiuseppe Di VitaSergi MaicasGiuseppe GambacortaSergio Alatorre-SantamaríaGiuseppe IacominoSergio E. Medina-CuéllarGiuseppe MangioniSergio Gómez-CornelioGiuseppe ManiaciSergio O. Serna-SaldívarGiuseppe ManninoSergio OrtizGiuseppe MartinoSergio Pérez-BurilloGiuseppe MecaSergio PflanzerGiuseppe MicalizziSergio RivaroliGiuseppe NatrellaSergio Torres-GinerGiuseppe PezzottiSerhat Sezai ÇiçekGiuseppe RussoSeri Intan MokhtarGiuseppe SpanoSerkan Serkan SelliGiuseppina AdilettaSerrano SaludGiuseppina CaraccioloSeung-Joo LeeGiuseppina MandalariSeung-Oh SeoGiuseppina ParpinelloSeung-Yoon OhGiustino TribuziSeveriano R. SilvaGiusy Rita CaponioSeverino ZaraGiverson MupambiSevgi̇n DiblanGjylije HotiSevim KoseGlaucia Braz AlcantaraSewa RamGlaucia Maria PastoreSewook OhGlayciane Costa GoisŞeyda BostancıGleidson Giordano Pinto De CarvalhoSeydi YıkmışGlen FoxSeyed Ali MoratazaviGlen Patrick FoxSeyed Enayat HashemiGloria Jiménez-MarínSeyed Hossein HoseinifarGohar KhachatryanSeyed Mohammad MazloomiGökçe Şeker KaratoprakSeyed Mohammad Taghi GharibzahediGokhan OzunerSeyed Saeid HosseiniGolfo MoatsouSeyed-Ahmad ShahidiGonca AlakSeyedeh MirpoorGonzalo Díaz-MenesesSeyyed Mehdi KhoshfetratGonzalo MejiaShafqat QaisraniGopal Lal KhatikShaghef EjazGopinathan H. MeletharayilShah ZamanGoran FrukShahab A. ShamsiGoran GajskiShahid AdeelGoran KusecShahla WunderlichGoran MitulovicShahzad IqbalGoran TrbicShaik Abdul HussainGordana ĆetkovićShailesh DaveGordon BarbaraShaimaa SelimGoreti BotelhoShalendra KumarGörkem ÖzülküShancen ZhaoGovindaraj Dev KumarShanmugam HemaiswaryaGraciel DiamanteShanthi G. ParkarGraeme GilliesShao-Wen HungGrant CampbellSharad UpadhyayGrant N. BennettSharmistha BarthakurGrazia Giuseppina PolitanoSharon PuleoGrażyna BudrynShashi Kishor PankajGrażyna Cacak-PietrzakShaun LeiversGrażyna Czaja-BulsaShayma Thyab Gddoa Al-sahlanyGrażyna Czyżak-RunowskaSheikh Safiul IslamGrażyna LewandowiczSheng FangGrazyna NeunertSheng ZhangGrażyna WejnerowskaShengbao CaiGreici BergamoShenghui CuiGreta AdamczykShengjie LiGreta FaccioSher AliGreta GaianiSherif EbadaGreta KrešićSherif MohamedGrethe HyldigSherlan Guimarães LemosGro Haarklou MathisenSheryl BarringerGrzegorz BartoszSheta M. ShetaGrzegorz KowalskiShigeaki UenoGrzegorz ZagułaShih-Chung WeiGuadalupe Virginia Nevárez-MoorillónShilin LiuGuang-Yuan HeShiling LuGuan-James WuShima SaffarionpourGuan-Jhong HuangShimaa AmerGuilherme FerreiraShiming LiGuillem CampmajóShing-Hwa LiuGuillermo Cristian Guadalupe Martínez ÁvilaShinjiro TachibanaGuillermo Fernando RegodónShintaro PangGuillermo Hugo PeraltaShiv Dutt PurohitGuillermo MoynaShivani PathaniaGuillermo PetzoldShivankar AgrawalGuillermo RipollShoukui HeGuillermo RodríguezShouwei WangGuizhao LiangShruti ShuklaGülay BaysalShuaishuai LiGulay OzkanShuang QiuGulcin YildizShuangping LiuGulyaim SagandykovaShu-Juan YuGulzar Ahmad NayikShunji KatoGuoxun ChenShunsuke FurutaniGuozhong ZhaoShuxun LiuGuozhou LiaoShu-Yao TsaiGupta VikasShyang-Chwen SheuGurunathan JayaramanSiamak ShiraniGurvinder Singh KocherSiddabasave B. B. GowdaGustavo Araujo PereiraSidonia MartínezGustavo Cabrera-BarjasSiemowit MuszyńskiGustavo Gonzalez-NevesSihan ChenGustavo PimentelSila KittiwachanaGustavo ZuñigaSilva Maria ManuelaGüzin KabanSilvani VerruckH. M. Prathibhani Chamidha KumarihamiSilvia LandiHack-Yeon KimSilvia LiscianiHadi Hashemi GahruieSilvia MarzocchiHadi PourjafarSilvia MatiacevichHadi RastinSilvia MironeasaHadis RostamabadiSílvia PetronilhoHae-Yeong KimSilvia PichardoHah Young YooSilvia VincenzettiHaidy Abdel Moniem GadSilvie BernatováHakan ArslanSilviu-Gabriel StoreHale Inci ÖztürkŠime MarcelićHalim HasseriSime UkicHalmuthur M Sampath KumarSimeon MinicHamada ElwanSimon C. AndrewsHamdy ShaabanSimon GregersenHammad BadarSimon Vlad LucaHammad JanSimona BenedettiHammad UllahSimona Catalina StefanHamza Youssef IsmailSimona ErricoHanae Naceiri MrabtiSimona JancikovaHande DemirSimona Lucia BavaroHang MaSimona ManHang QiSimona SagonaHanifah Nuryani LioeSimona ViolinoHanna BaranowskaSimone AngeloniHanna Górska-WarsewiczSimone BellucoHanna KowalskaSimone CavaleraHanna Maarit KoivulaSimone FattoriniHanne Christine BertramSimone GiacosaHanne Kristine SivertsenSimone MazzuttiHans DagevosSimone Monteiro SilvaHan-Seok SeoSimone VincenziHans-Joachim KnölkerSinh Dang-XuanHans-Joachim SchüllerSiniša MarkovHany Abdel Satar ElkashefSiniša SrečecHany G. Abd El-GawadSiong Meng LimHany H. ArabSiqi LiHao LiSirirat DeeseenthumHaoan ZhaoSirivan AthikomkulchaiHari Prasad DevkotaSirma YeginHarish HandralSishir K. KamalapuramHarish KumarSit NandanHarish Kumar ChopraSiti KamalHasan JaliliSiti Kholijah Abdul MudalipHasan S. A. JawadSivakamavalli JeyachandranHaşim AltanSlađana DavidovićHasim KelebekSlađana ĐurđićHasmadi MamatSladjana JevremovicHassan Ahmadi GavlighiSladjana RakitaHassan BarakatSlaven JurićHassan Karimi-MalehSlavica StankovićHassan ZafarSlavisa StajicHatamian-Zarmi AshrafalsadatSlavomír MarcinčákHazem RamadanSławomir KulaHazuki TamadaSlawomir OrzechowskiHe ZhuSlim AbdelkafiHeba AbdelrazekSlim SmaouiHeba AhmedSmaoui SlimHeba ElgizawySmaragda SotirakiHebah Al UbeedSnežan Mladenović DrinićHéctor Aarón Lee-RangelSnežana ŠkaljacHector B. Escalona-BuendiaSnježana P. KazazićHedia Manai-DjebaliSobhy El-SohaimyHeena SharmaSofia AgriopoulouHefei ZhaoSofia GuedesHeikki VapaataloSofia MitakouHeinrich W. Du PlessisSofia Moran-RamosHejun WuSofia ReisHelen AguSofie ØstergaardHelen CollinsSohail QaziHelen OnyeakaSoheil SobhanardakaniHelen RogersSol García-GermánHélen RossetoSol ZamuzHelena Maria André BoliniSolmaz SaremnezhadHelena SilvaSolomiia KozachokHélène AuthierSomaris E. QuintanaHélène Licandro-SérautSomdip DeyHengyi XuSon Dong-JuHenrike FischerSonia BarrosoHenrique ReboredoSonia LaneriHeping CuiSónia PedroHerbert L. MeiselmanSonu SharmaHerbert Michael HeiseSoo Im ChungHerlina MartaSoo Jin YangHerman ViorelSook Chin ChewHermann NirschlSophie HiekeHerminia DomínguezSophie NuttenHerminia Vergara PérezSophie PracheHernández HerreroSophie Thetiot-LaurentHerwig BachmannSoraia I. FalcãoHesamaddin Shirzad-AskiSoraya PazHesham MoustafaSoraya Paz MontelongoHettie SchonfeldtSorin Marius AvramescuHicham HarharSotirios EkonomouHideaki ShigaSotirios KakabakosHileia SouzaSotiris GayialisHilton DeethSoudamani SinghHira SinghSpiros ParamythiotisHirac GurdenSrigopal SharmaHiroaki GotohSrikanth KarnatiHiroaki KitazawaSrinath PashikantiHiroko X. KondoSrinivas KoppuHironori HondohSrinivasan RamalingamHiroshi KikukawaStanislau BoguszHiroshi KitagakiStanislav KotlyarovHiroshi MatsufujiStanislava IvanovaHiroyasu MoriStanisław KaliszHiu-Yee KwanStanisław KowalskiHodaka SuzukiStanisław MlekoHong WangStavros KalogiannisHong ZhangStavros LalasHongbing FanStavros PlessasHong-Duck KimStefan G. DragoevHong-Jhang ChenStefan ToepflHongjie DaiStefania AlbrizioHongjuan LiStefania BarzaghiHongshun YangStefania CesaHongtao LeiStefania ChironiHong-Ting LinStefania ContiHong-Ting Victor LinStefania IamettiHongwei WangStefania MarcheggianiHongwei XiaoStefania MoramarcoHongxia WangStefano AmalfitanoHooi-Leng SerStefano FornasaroHoracio BachStefano MassagliaHorst-Christian LangowskiStefano PiazzaHosam El-AnsaryStefanos LeontopoulosHossein AzadiSteliana RodinoHossein EslamiStella OrdoudiHossein HaghighiStephan SommerHouda BerradaStephan WalchHoussam AbouloifaStéphane BalayssacHrotkó KárolyStephanie Archer-HartmannHrvoje PavlovićStéphanie DudonnéHsiao-Ping ChangStephanie Rodríguez BesteiroHsin-Tang LinStephen DevereuxHsu Yang LinStephen InbrajHuaiwen YangStephen J. JamesHuaixiang TianSteva LevicHuaiyou WangStrzemski MaciejHua-Jun FanStyliani MinoudiHualu ZhouStylianos ExarhopoulosHui YanSubhadeep ChakrabartiHui-Hui XiaoSubhankar SinghaHuipeng LiangSubhash BabuHujun XieSudarsan MukhopadhyayHulya YalcintanSudeshna SahaHuma AinSudhanshu JoshiHumberto BizzoSueli RodriguesHumberto González-RíosSujogya Kumar PandaHumberto Hernández SánchezSukamal SarkarHung N. MaiSukhvinder SinghHung-Ju LiaoŞükrü KalaycıHusam YounesSulayman OladepoHusnul Azan TajarudinSultan Arslan-TontulHye Won KimSuman AlishettyHyun-Jae ShinSumeet GuptaHyun-Jung ChungSun Moon KangHyun-Wook ChoiSuncica BeluhanHyun-Wook KimSundaramoorthy HaripriyaI. M. ChernukhaSuneetha VuppuIain A BrownleeSung Jun ChoIan JensonSung Ki LeeIan M. SimsSunghyup Sean HyunIbrahim A. NaguibSungyong ChoiIbrahim AlsohaimiSung-Yong HongIbrahim F. RehanSunita ChamyuangIbrahim GülserenSunita MeenaIbrahim KhalifaSuresh VeeraperumalIchiro TatsunoSurinder SinghIck Soo KimSurya PaudelIda Bagus Agung YogeswaraSusana Anahi DandlenIdoia PostigoSusana C. RibeiroIdolo IfieSusana Caldas FonsecaIftikhar Ali KhanSusana FiszmanIftikhar Hussain BadarSusana M. CardosoIga NehringSusana Santos BragaIga RybickaSusana SilvaIgnacio Peñalosa-CastroSusana SimalIgor Alexandre Da Silva DiasSusanna BurattiIgor AlmeidaSusanna Salminen-PaateroIgor HernandezSushil MiddhaIgor LoskutovSusumu MuroyaIgor LukićSutee WangtueaiIgor Piotr TurkiewiczSuzana Caetano Da Silva LannesIgor PravstSuzana MaliIgor TomasevicSuzana Rimac BrnčićIhab ShigidiSuzanne KalbIhsan HamawandSven KarlovićIjung KimSvetlana DerkatchIkram AliSvetlana M. MomchilovaIl Soo MoonSvetlana N. SytovaIlaria MarchioniSvetlana NikoličićIlda CaldeiraSvetlana SimovaIlenia TinebraSvetoslav TodorovIlias GiannenasSwarna JaiswalIlija DjekicSyed Amir AshrafIlona GórnaSyed Ammar HussainIma WijayantiSyed Bilal HussainImad KansauSyed Mithun AliIman ZareiSyed Zameer HussainImran AliSylwester CzaplickiImran ImranSylwester MazurekIna JasutieneSylwester ŚlusarczykIndira T. KudvaSylwia BalutaIndrikis KramsSylwia LewandowskaInese FilipovaSylwia OkońInga CiprovičaSylwia Onacik-GurInge-Willem NoordergraafSylwia Zakowska-BiemansIngrid Rodríguez-BuenfilSzabolcs FogarasiIngrida MažeikienėSze Ying LeongIngunn BergetTabussam TufailIngvar SvanbergTae Kon KimInken Christoph-SchulzTae-Kyung KimInmaculada FrancoTae-Woon KimInmaculada GómezTafadzwa KasekeInmyoung ParkTai-Chia ChiuInna SimakovaTaikei SuyamaInnocence HarveyTakafumi ItohInsuck BaekTakashi OhshiroInthawoot SuppavorasatitTakashi UebansoIoan GrozescuTakashi YoshidaIoana BalanTakayuki MizunoIoanna ChinouTakuji TanakaIoanna NtaikouTalha DemirciIoannis A. GiantsisTalwinder S. KahlonIoannis K. KarabagiasTamara DapcevicIoannis KourkoutasTamara PapovićIoannis MourtzinosTamara TavoloniIoannis RoussisTamer Mohammed El-MesseryIoannis VagelasTamer ShehataIoannis ZabetakisTammara SomaIon SanduTaner SarIonica CoțovanuTania Cecilia MihaiescuIonica OncioiuTânia FerreiraIppolito CameleTania Maria Grazia SalernoIrena BarukcicTanja BogdanovićIrena Brčić KaračonjiTanmay SarkarIrena Budić-LetoTantawan PirakIrene CaffaTao FengIrene DiniTao HuangIrene Gómez-CruzTao WangIrene KamenidouTaotao DaiIrene RomeroTapan SarkarIrene StefaniniTarek Ben HassenIreneusz KapustaTarek SolimanIrfan A. RatherTareq M. OsailiIrfan AliTariq AliIrfan ErolTariq AzizIrfan RatherTariq IsmailIrina FierascuTarja K. NevanenIrina GushinaTarun Kumar DuaIrina IelciuTarun UpadhyayIrina MaslennikovaTatiana BojňanskáIrina TsitkoTatiana Carlesco Dos SantosIrineu BatistaTatiana Souza PortoIrini DoytchinovaTatjana KošmerlIrini F. StratiTatjana M. MajkićIris PlioniTatsuro OhiraIrmanida BatubaraTatsuya MoriyamaIrwin Alencar MenezesTaweepol SuesutIsa FusaroTeleky Bernadette-EmokeIsaac AmoahTeneva DesislavaIsaac RodríguezTeobald KupkaIsaac Yves Lopes MacêdoTeodora Emilia ColdeaIsabel BeloTeodora IvanovaIsabel CaballeroTeresa Ayora-TalaveraIsabel FernandesTeresa BergholzIsabel FerreiraTeresa M. BergholzIsabel PrietoTeresa MadureiraIsabel SayagoTeresa MougaIsabella EndrizziTeresa Rubio-TomásIsabelle Verdier-MetzTeresa SemedoIsam Tawfik KhadimTeresa ValdiviessoI-Shiang TzengTeresa WitczakIsidora RadulovTerhi PohjanheimoIsmail IshamriTessa R. EnglundIsmail M. M. RahmanThaís Matsue UekaneIsmail YenerThais MilessiIsoken IgbinosaThais Suzane Milessi EstevesIsrael MuñozThalia TsiakaIsrael S. IbarraThangamuthu Mohan DasIssis Quispe-FuentesTheodoros VarzakasIstván DalmadiTheofania TsironiIstván EgerszegiTheofylaktos MassourasIstván KomlósiThi Minh Phuong NgoIstvan PelczerThibault NideletI-Ta LeeThierry AussenacÍtala M. G. MarxThierry ClavelItziar ChurrucaThierry TranItziar RuisánchezThilahgavani NagappanIuga MadalinaThiruventhan KarunakaranIuliana AproduThomas BintsisIván Adrián García GaliciaThomas GoudoulasIvan BestThomas Iain DugmoreIvan BobrinetskiyThomas Matthew TaylorIván CarreraThomas RégnierIván Carrillo-BerdugoThorsten KraskaIvan LesovThuan-Chew TanIvan Luzardo-OcampoThumnoon NhujakIvan M. SavicThunnop LaokuldilokIvan ShorstkiiThyago NepomucenoIvan VnučecTiago NazarethIvan ZlatanovićTiancai LiuIvana BuljetaTiberia Ioana PopIvana GadjanskiTiberius BalaeşIvana Generalić MekinićTiboni MattiaIvana GiangriecoTicuta Negreanu-PirjolIvana IvicTilo LuebkenIvana LončarevićTim MaischIvana NikolićTimofey PylaevIvana PajčinTimothy M. EschleIvana PolišenskáTimothy SnowdenIvana Savic GajicTimothy TseIvana Sredović IgnjatovićTina Gulin-SarfrazIvana T KarabegovicTina KostkaIvana V. SofrenićTingting BuIvayla DinchevaTingting ZouIvelina DessevaTirawat RairatIveta KlojdováTiziana BacchettiIvica KosTiziana De-MagistrisIwona GientkaTiziana Di RenzoIwona GolonkaTiziana PepeIwona GrabowskaTobi FadijiIwona KonopkaTobias GorisIwona KowalskaTodor BogdanovIwona ŚcibiszTodor TodorovIwona Wojtasik-KalinowskaTodor VasiljevicIza F. Peréz-RamírezTolulope Joshua AshaoluIzabela FeckaTom GillIzabela Jasicka-MisiakTomas BaležentisIzabela MichalakTomáš HájekIzabela Szymczak-PajorTomás HerraizIztok KosirTomasz BajIztok PrislanTomasz CebulakJ. A. PereiraTomasz CzajaJ. B. WilliamsTomasz DaszkiewiczJ. GawałekTomasz FlorowskiJ. Jesús Vargas RadilloTomasz JakubowskiJ. L. Gómez ArizaTomasz KnapowskiJ. P. BowmanTomasz LesiowJaber NasiriTomasz Pawel CzajaJacek AndrzejewskiTomasz SawickiJacek BaniaTomasz SkrzypekJacek E. NyczTomasz SzablewskiJacek LewandowiczTomasz SzymborskiJacek ŁyczkoTomislav BosiljkovJacek OsekTomislav KaražijaJacinta Collado-GonzálezTomislav MikušJack YangTomislav TostiJackline Freitas Brilhante De Sao JoseTomislava Vukušić PavičićJacob FolzTommaso FrioniJadwiga HamułkaTommaso R.I. CataldiJadwiga ManiewskaTomohiko KomatsuJae Kwang KimTomoya YoshinariJae-Youl ChoTong SunJae-Young JeTonna A. AnyasiJagadeeswara Reddy VennapusaTony MutukumiraJagdish KhubchandaniTorey LooftJagoda O. SzafrańskaToribio LauraJaime Moisés RomeroTorsten BohnJakkrawut MaitipToshihiko ShojiJaksuma PongsetkulToshikazu MorishitaJakub BabolTran Dang XuanJakub DybasTrinidad Pérez PalaciosJakub SzperlikTripti AgarwalJames K. H. FangTruongngoc MinhJames T. TambongTshimangadzo MunondeJamshaid HussainTsun-Thai ChaiJan BártaTualar SimarmataJan BedrníčekTuba BaygarJan MieszkowskiTümay TemizJan SýkoraTuncay YılmazJan Willem Van Der KampTurid RustadJana CopikovaTusneem KausarJana Šic ŽlaburTuyen KhaJana ŠtefánikováUeli Von AhJana StofilovaUelinton PintoJanak KhatiwadaUgur CakilciogluJane O'SullivanUjjwal LayekJanet Adeyinka AdebiyiUlrich KulozikJanet RiedlUlrike Van Der SchaafJaneth M. Ventura-SobrevillaUma Devi PalanisamyJang Eok KimUmair AhsanJanina GospodarekUmapathi ReddicherlaJanine Fleith De MedeirosUmar Ijaz AhmedJanitha WanasundaraUmesh Basanagouda DeshannavarJaromir LachmanUmmat VirujaJaroslav TóthUndine LehmannJarosław L. PrzybyłUpaka RathnayakeJaroslawa RutkowskaUr Rehman NaveedJasenka Gajdoš KljusurićUrban BrenJaslyn LeeUroš ČakarJasmina VitasUroš M. GašićJasna Čanadanović BrunetUrška BlaznikJasna DjordjevićUrška RozmanJasna MastilovićUrszula Gawlik-DzikiJasna MrvčićUrszula Krupa-KozakJasna NovakUrszula SamotyjaJasna TwynstraUrszula SzymanowskaJasneet GrewalUrszula TylewiczJason SawyerUrszula ZarzeckaJaswinder SinghUta SchnabelJatin KalitaUtai KlinkesornJau-Shya LeeUte SchröderJavad HeshmatiVagner De Alencar Arnaut De ToledoJavier CarballoVahid FarzanehJavier Cremades UgarteVaibhav A. MantriJavier EnrioneValdas JakštasJavier Germán Rodríguez-CarpenaValdir VeigaJavier IllescasValentin RauhJavier Linares-PasténValentina CanutiJavier MateoValentina DomeniciJavier SaurinaValentina FanelliJavier UrracaValentina GiovenzanaJayalakshmi AyyavooValentina LacivitaJayashree ArcotValentina MarassiJayasimha Rayalu DaddamValentina Maria MerlinoJayesh J. AhireValentina MeliniJayne BockValentina NarducciJean Daniel CoissonValentina NikolićJean SavoieValentina RizzoliJean-Denis TroadecValentina ZhurikhinaJeanine AmmannValentine BordierJean-Luc PutauxValeria BorsellinoJean-Marie CardebatValeria Rachela VillellaJean-Marie LaplaceValeria RizzoJeannine MaraisValérie GaudinJean-Philippe RalValerija DunkićJean-Xavier GuinardValerio GiacconeJee-Seon ShimValerio ManippaJeff WoodValério Monteiro-NetoJefferson Romáryo Duarte Da LuzValeriu V. CoteaJelena KruljVan Giau VoJelena MijalkovićVanda PereiraJelena MuncanVanesa RomeroJelena TomićVanessa CaprilesJelena VladićVanessa VieiraJenifer SantosVangelis EconomouJennifer Martins NoroVânia Borges FerreiraJens StaalVania PatroneJenshinn LinVania Urías-OronaJenyffer Campos GuerraVania Zanella PintoJeonghee KimVanja M. TodorovićJérémie ThéolierVanja SeregeljJernej OgorevcVanja TadićJerome MounierVarit SrilaongJer-Yiing HoungVaroujan YaylayanJerzy GrabińskiVaruzhan SarkisyanJerzy JuśkiewiczVarvara AndreouJessica ZampolliVasia S. MavrogianniJessy Van WykVasil SimeonovJesus BalsindeVasilevskaya EkaterinaJesús Eduardo Quintanilla-LópezVasiliki KachrimanidouJesús Fernando Ayala-ZavalaVasiliy Yu. TsygankovJesús Gilberto Arámburo GálvezVassilis AthanasiadisJesus Olivero-VerbelVassilis DourtoglouJette Feveile YoungVassya BankovaJeyan MosesVazhiyil VenugopalJheng Hua LinVeerachandra K. YemmireddyJhih-Ying CiouVenkateswarlu SunkesulaJi Yeon KimVenugopal GangurJia SongVera AmicarelliJian ZhangVerónica Carrasco-SánchezJiang ZhengVeronica GinaniJianhua ZhuVeronica Sanda ChedeaJianke LiVeronica SberveglieriJianquan KanVerónica SeguraJie GaoVeronika BarišićJie Hong ChiangVeronika SeleJie SunVéronique BoscJih-Jung ChenVéronique Verrez-BagnisJim MclauchlinVesa Cosmin MihaiJin YueVesela ShopskaJinchao LouVeselina PanayotovaJinchuang ZhangVesna A. PerićJing MaVesna IlićJing XuVesna JovanovićJinghua WangVesna Tumbas ŠaponjacJingli XieVibeke OrlienJingming NingVicente Espinosa-SolisJingran BiVicente M. Gómez-LópezJingyu ChenVicente Serna-EscolanoJing‐Yueh JengVictor Benno Meyer-RochowJinhe BaiVictor LaderoJin-Hee LimVictor RodovJinhong WuVíctor Ruiz-Valdepeñas MontielJinhua ChengVida JuozaitienėJinling WangVida ŠimatJinxuan CaoVidmantas BendokasJinyao ChenVignesh MuthusamyJirawat YongsawatdigulVijay ChhetriJiunn-Ming SheenVijay Kumar Reddy SurasaniJixian ZhangVijay VictorJiyoon YiVijaya Anand ArumugamJoan M. KingVikas KumarJoan VallèsVikash Chandra RoyJoana CostaVi-Khanh TruongJoana Inês Bastos BarbosaVikram PatialJoana PintoViktoria ZettelJoan-Francesc Fondevila-GascónVilma KaškonienėJoanna BierłaVilma QuitralJoanna BrysVincenza DolceJoanna ChmielewskaVincenzo CastelloneJoanna DąbrowskaVincenzo GerbiJoanna GruszczyńskaVincenzo MarcotrigianoJoanna GrzelczykVincenzo TufarelliJoanna HarasymVinicius Silva CastroJoanna KaszubaVinit RajJoanna Kawa-RygielskaVinko KrstanovićJoanna KlepackaViola GalliJoanna Le Thanh-BlicharzVioleta Ivanova-PetropulosJoanna LeszczyńskaVioleta RuiperezJoanna Małgorzata KrukVioleta T. Pardío-SedasJoanna NowosadViorica ChirilaJoanna OrzelVira I. LubenetsJoanna PawłatVirgílio De Carvalho D. AnjosJoanna Piepiórka-StepukVirgínia Alice Cruz Dos SantosJoanna Pławińska-CzarnakVirginia Celia ResconiJoanna PodlasińskaVirginia Yagüe JiménezJoanna Rachtan-JanickaVishal KumarJoanna ZarzynskaVisnja StulicJoanna ZembrzuskaVita De StefanoJoanna Zochowska-KujawskaVitaly SchetininJoão CotasVitor Andre Silva VidalJoão DiasVitor Augusto GarciaJoao Filipe Alberto LopesVítor João Pereira Domingues MartinhoJoão Guilherme De Moraes PontesVittoria RaimondiJoão LimaVivian FeddernJoão M. LeçaViviana ManzulliJoão Paulo WiniarskiViviana P. JofreJoão Rodrigo SantosVivienne ReeveJoaquina PinheiroVladana GrabezJochen VestnerVladimir FilipovicJoel D. MainlandVladimir KalininJohannes De BruijnVladimir KnyazJohannes DelgadoVladimir PacutaJohannes StraussVladimir TomovicJohn BeaulieuVladimir-Lucian EneJohn C. PetersVlasios GoulasJohn CarragherVojislava BursicJohn GrigorVojka BabićJohn KapolosVolkan Arif YılmazJohn KennyVolkan OkatanJohn M. ThompsonVuk B. ĐorđevićJohn MackayWael ElkotJohn Masani NdukoWafaa ElkadyJohn MisasiWageh DarwishJohn QuiñonesWahauwouele Hermann CoulibalyJohn SamelisWahyudi Budi SediawanJohnson O. EsuaWai San CheangJoice Tom JobWajaht A. ShahJolanta CieślaWalaa NegmJolanta GawalekWaleed El-GhareebJolanta KowalskaWaleed S. AlharbiJolanta WawrzyniakWalid ElfallehJonathan PerrinWalter F. SchmidtJonathan S. TamWan Abd Al-Qadar Imad Bin Wan MohtarJonathan Sabaté Del RíoWan RosliJong-Whan RhimWanda WadasJoo Shun TanWanderson SilvaJoon-Young JunWang WenlongJordan W. BeaverWaqar AhmadJordana Rivero VieraWaras NurcholisJorge A. ZegbeWataru MizunoyaJorge Alberto Sanchéz-BurgosWayne JiangJorge Antonio Sánchez-MolinaWee Yin KohJorge Enrique Wong PazWei LuJorge EstévezWei QuanJorge FreitasWei XuJorge García-HernándezWei YuJorge Geovanny Figueroa HurtadoWei ZhouJorge Herman BehrensWei-Lun HungJorge LagoWeiping XuJorge Luís Victória BarbosaWeiwei ChengJorge PereiraWeiwei CuiJorge ReinheimerWelder A. BaldassiniJorge SaraivaWellison Jarles Da Silva DinizJorge Velásquez-CockWen MaJorge Verdú-AndrésWen Shyang ChowJørgen J. LeisnerWenchao YangJosafat Ezquerra-BrauerWen-Chieh SungJosé A. Cano BuendíaWen-Chien LuJosé Agustín Tapia HernándezWendy FrancoJose Angel Pérez-AlvarezWenhang WangJosé António CoutoWenjiang DongJosé António De Oliveira E SilvaWenjie ZhengJosé Antonio Guerrero-SolanoWen-Jun LiuJosé Ascención Martínez ÁlvarezWenli WangJosé Carlos Tavares CarvalhoWenqian ChenJosé Cleiton Sousa Dos SantosWenxin SongJosé De Jesús Manríquez-TorresWeronika Korpysa-DzirbaJosé De Jesús Ornelas-PazWidiastuti SetyaningsihJosé De Jesús Zazueta-MoralesWiesław PrzybylskiJosé E. MesoneroWijitha SenadeeraJosé E. ZapataWiktor BerskiJosé Enrique Torres VaamondeWilliam PotterJosé Fernando Rinaldi AlvarengaWilliam R. NewsonJosé Francisco Segura PlazaWilliam SganzerlaJosé Hipólito Isaza MartínezWilma Maria Coelho AraújoJose Juan Buenrostro FigueroaWilman CarrilloJosé Juan Rodríguez-JerezWilmer Carvache-FrancoJosé Laerte NörnbergWioleta Chajȩcka-WierzchowskaJosé Luis GuzmánWioletta BielJosé Luis Hernández-HernándezWioletta BłaszczakJosé Luis Montañez-SotoWioletta DrożdżJosé Luis RiverosWioletta Zukiewicz-SobczakJosé Luis Rodríguez GutiérrezWirawan Dony DahanaJosé Luiz De Brito AlvesWirginia Kukula-KochJose M. MirandaWirote YouravongJosé M. OliveiraWissem Aidi WannesJosé Manuel Aguilar GarcíaWissem MnifJosé Manuel Pais-ChanfrauWitold SzczurekJosé Manuel Rodríguez-NogalesWoei Jye LauJosé Miguel PestanaWojciech FabianowskiJosé MuñozWojciech KochJosé PinelaWojciech KolanowskiJose RamosWojciech PiekoszewskiJose Ros-LisWon Y. LeeJosé Santos García AlvaradoWong Yong FooJose Sebastián Velázquez BlázquezWoo-keun KimJose Sousa CamaraWorawan PanpipatJosef KameníkXavier MontanéJosefa TolosaXi YuJosefina Villanueva RodríguezXianbing XuJosemar Gonçalves De Oliveira FilhoXiang LiJosep ComaposadaXiang XiaoJosep Valls FonayetXiangyang ZhouJoseph ArulXianjiang LiJoseph KannerXiao ChenJoshua E. MuscatXiao HeJoshua O’HairXiao Li ShenJosivanda P. GomesXiaobo HeJouko PeltonenXiaobo ZouJovana KojićXiaochang XueJovana KosXiaodan DingJovana KovacevicXiaofang ZengJovana PetrovićXiaofen DuJovana RajkovicXiaojing LengJovana VundukXiaojuan MaJovanov PavleXiaolong WangJovica LončarXiaolun SunJoya KemperXiaomei BieJoyce SilvaXiaopeng HuangJozef ČaplaXiaopeng TangJozef SowinskiXiaoshan LiJózsef CsanádiXiaoyong ZhangJuan A. CentenoXiaoyun LiJuan A. Morales-RamosXiaxia DiJuan B. CalancheXijun LianJuan Carlos Cuevas BernardinoXin HuangJuan Carlos Hernández-GonzálezXin ZhangJuan Carlos López-LinaresXingyi JiangJuan Carlos Romero-BenavidesXingyu ShenJuan Darío Ríos MeraXinping LinJuan José Martínez NicolasXin-Tang LinJuan José Ramos-RodríguezXinyou CaoJuan Manuel Ramón JerónimoXiomara-Fernanda Quinones-RuizJuan Manuel TiradoXiufang BiJuan MejutoXiuxiu SunJuan Monzó-CabreraXiuying QiaoJuan MunizXochitl Aparicio-FernándezJuan Pablo Fernández-TrujilloXochitl Ruelas-ChaconJuana FríasXristo ZarateJuan-Rodrigo Bastidas-OyanedelXuan ZhuJudit S. TóthXuepeng LiJudita LidikováXuequn PangJuhee AhnXuewu GuoJulė JankauskienėXuezhi BiJulia DurekYafeng ZhengJulia PetersonYalda Rahbar SaadatJulián Andrés Gómez-SalazarYan CampbellJulian BlascoYan LamJuliana Aparecida Correia BentoYan LiJuliana Azevedo Lima PalloneYan ZhouJuliana CotabarrenYanbo WangJuliana Morales-CastroYanchao WangJuliana VillasanteYang BiJuliane WelkeYang FuJuliano GaravagliaYang HuJuliano UczayYang LinJulie JonesYangyong LvJulieta Domínguez-SoberanesYanjiao LiJulio Cesar Dos ReisYanjun ZhangJulio Montes-ÁvilaYanli ZhouJulio Nogales-BuenoYannick WeesepoelJulio Parra-FloresYanty Noorzianna Abdul ManafJulio Plaza DíazYanwen XiongJun DangYanyan WuJun LiYaosong WangJun WangYaowapa LorjaroenphonJun WatanabeYaowared ChulikhitJun YoshinagaYaoyao PengJunfeng XuYaqin ChaiJunfeng ZhaiYasir IqbalJung Eun KimYasuhiro KogaJung-Heun HaYasutaka ShigemuraJung-Seok ChoiYasuyuki MakiJun-Hui ChoiYe JinJun-Ichi KadokawaYeimmy Peralta-RuizJunsoo LeeYeongbae ChoeJuraj MajtanYeşim ÖzoǧulJurga BūdienėYeung Joon ChoiJuristo FonolláYheni DwiningsihJuscelino TovarYhiya AmenJustyna BrzezichaYi ChenJustyna Brzezicha-CirockaYilmaz UcarJustyna CybulskaYimin QinJustyna GrabskaYimin ZhangJustyna Krzyżanowska-KowalczykYing ChenJustyna LiberaYing JiJustyna Rosicka-KaczmarekYing Ping ChangJustyna ŻulewskaYing YangK. B. HebbarYining JinK. Singhachuit AchuitYinji ChenK. Thomas KlassonYiqi ZhangKa LiYi-Qi ZhangKa Wan LiYiwen ZhangKabilan VelliyagounderYlva Mattsson SydnerKacie HoYoana KiselovaKacper ŚwiechowskiYogesh KhetraKagan KermanYogesh KumarKah Hui WongYohei ShirakamiKai Min YangYolanda Moliner MartinezKai WangYolanda Paola MaturanoKai ZhangYolanda Terán-FigueroaKai ZhongYolande T. R. ProrogaKaihui LuYongfeng LiuKaiqiang WangYonghua LiKaiser YounisYonghuan YunKajetan DabrowaYongjun WeiKajetan MüllerYongliang ZhuangKaliyan BarathikannanYongquan XuKaloyan PetrovYong-Suk KimKalu Kapuge Asanka SanjeewaYosef Al ShoffeKamal Ahmad QureshiYoshimi SeidaKamal KansouYoshio MakinoKamel Abd-ElsalamYoshitaka NakayamaKamil DrabikYosra A. HelmyKamil JurowskiYouenn LohéacKamil PiwowarekYouling XiongKamila BorowiecYoung Hoon JungKamila RachwalYoung Hun JinKandhasamy SowndhararajanYoung Min ChoiKandi SridharYoung-joo AhnKaneda HirotakaYousef NaserzadehKang-Mo KuYouzhong LiuKanika KhannaYu GeKankan ZhangYu ToyodaKannappan ArunachalamYu XiaoKaren Ildico Hirsch-ErnstYu YangKaren S. ByrdYu. F. ZuevKarim El-SabroutYu. S. SidorovaKarin HufnaglYu. V. PisarevskyKarin MandlYuan YuKarin SchroenYuan-Shuai LiuKarin SchwaigerYuanzhong WangKarina BatistaYucai HeKarine CharryYuhao ZhangKarl R. MatthewsYuh-shuen ChenKarla Bigetti GuergolettoYu-Hsiang LeeKarlo JuricaYuhua WangKarna RamachandraiahYuki TakaiKarol SteckiewiczYuliya HrynetsKarolina A. Wojtunik-KuleszaYumin DaiKarolina Brkić BubolaYun XuKarolina CelejewskaYung-Fu HuangKarolina JakubczykYung-Kai LinKarolina JurkowskaYunia García TejedaKarolina KrólikowskaYunping YaoKarolina LabusYuntao DaiKarolina Matej-ŁukowiczYurong GuoKarolina PawlakYus Aniza YusofKarolina PyciaYutaka ItabashiKarolina RatajczakYuthana PhimolsiripolKarolina Szewczyk-GolecYuwares MalilaKarolina SzulcYu-Wei ChangKarolina WiszumirskaYuwei YuanKarolina Wojtunik-KuleszaYuyang HuangKaroly HebergerYu-Yi ChanKarthik Sajith BabuYuyue QinKarthikeyan SubburamuYuyue ZhongKarthikeyan VenkatachalamYuyun LuKashif AkramYves PouliotKashif AmeerYves-Jacques SchneiderKatarina LukićYvonne GranfeldtKatarina MihajlovskiZaara SarwarKatarina SmiljanićZachary S. ClaytonKatarina SmilkovZafer CeylanKatarína ŠuchováZafer ErbayKatarina VeljovićZahid NaseerKataryne Árabe Rimá De OliveiraZahoor Ul-HassanKatarzyna BączekZahra GharariKatarzyna BanachZahra HadianKatarzyna CzyżZakaria Hossain ProdhanKatarzyna JandaZakaria TazartKatarzyna Kajak-SiemaszkoZamantha Escobedo-AvellanedaKatarzyna Marciniak-LukasiakZanda KrūmaKatarzyna PobiegaZaneta Czyznikowska-BalcerakKatarzyna RatuszŻaneta PolkowskaKatarzyna RybakZapryana R. DenkovaKatarzyna ŚliżewskaŽarko KevrešanKatarzyna ŚmiecińskaZbigniew KobusKatarzyna Sułkowska-ZiajaZbigniew OtrembaKatarzyna ŚwiąderZdenka KádekováKatarzyna SzewczykZeev WiesmanKatarzyna TkaczZehra Hajrulai MusliuKatarzyna WaszkowiakZeki Atıl BulutKatarzyna Wolny-KoładkaŽeljka MesićKatell Peoc'hŽeljko AndabakaKateřina DadákováZeljko KnezKaterina KotzampassiZemede AsfawKatherene O. B. AnguahZenaida SaavedraKatherina SiewertZengqian ShiKathia Jimenez MonroyZengwang GuoKathrine BakZeynep Tacer CabaKathy LewisZgoła-Grześkowiak AgnieszkaKatia BarbaroZhanibek YessimbekovKátia FernandesZhaojun WeiKatia PetroniZhaowei ZhangKatie Parish-VirtueZhaoyang DingKatieli Da Silva Souza CampanholiZhen Xing WangKatrin KomolkaZhengwei HuangKatrin ZanderZhengyun WuKavita SharmaZhenjia ZhengKaWang LiZhenzhou ZhuKazeem AdeyemiZhida SunKazeem AlayandeZhihong SunKazue IshitsukaZhijie BaoKazuya MorimatsuZhili WanKe WangZhimin XuKegan Romelle JonesZhixiang ZhangKeith WarrinerZhixiong ZengKenji MaehashiZhi-Yong GongKenji SatoZhiyong HuangKenneth C. OlsonZhiyun ZhangKenneth SöderhällZhong HanKenneth W. McMillinZhong ZhangKerry WilkinsonZhongjiang WangKertész IstvánZhong-quan SuiKeshun LiuZhongwei ZouKevin KantonoZhongxiang FangKevin Mis SolvalZhongyang DingKevser KaramanZhuang GuoKeyu TaoZhuangli KangKeyvan PakshirZhuo WangKhawar SiddiquiZhuoyan HuKhongsak SrikaeoZiarno MałgorzataKhuda FazliZi-Chao WangKhursheed ShiekhZienab F. R. AhmedKhushwant Singh BhullarZifan WanKi Hwan ParkZinash A. BelayKi Hyeong ParkZivile TarasevicieneKianoush Khosravi-DaraniŽivoslav TešićKieu The Loan TrinhZlatin ZlatevKi-Jong RheeZlatko SvečnjakKijung PaengZofia ZydlikKim JanssensZoltán KókaiKim Lambertsen LarsenZonghang ZhangKim StanfordZongmei GaoKinga GawlińskaZongshuai ZhuKinga TopolskaZoran ZekovićKiri McCombZorana MiloradovicKishneth PalanivelooZorba Josué Hernández-EstradaKit Leong CheongZorica StojanovićKitipong AssatarakulZormy Nacary Correa PachecoKittibunchakul SuwapatZsuzsanna SandorKiyosumi HoriZuamí Villagrán-de La MoraKlaudia MasztalerzZuhaibfayaz BhatKlaus DürrschmidZul Ariff Abdul LatiffKodiveri Muthukaliannan GothandamZuzana GajdosechovaKok Heng SoonZuzana JurasekovaKomwit SurachatZuzanna Żołek-TryznowskaKong Shun Ah-HenZvjezdana Findrik BlaževićKoni Grob



